# Salivary microbial meta-analysis reveals gender differences in oral microbiota, core microbiota, and molecular markers

**DOI:** 10.3389/fcimb.2026.1796284

**Published:** 2026-04-15

**Authors:** Qixiang Yuan, Yifan Zhang, Zilu Wang, Songnian Hu, Xudong Liu, Zilong He

**Affiliations:** 1 School of Engineering Medicine, Beihang University, Beijing, China; 2 State Key Laboratory of Microbial Resources, Institute of Microbiology, Chinese Academy of Sciences, Beijing, China; 3 Laboratory Animal Research Facility, National Infrastructures for Translational Medicine, Institute of Clinical Medicine, Peking Union Medical College Hospital, Chinese Academy of Medical Science and Peking Union Medical College, Beijing, China; 4 Key Laboratory of Big Data-Based Precision Medicine, Beihang University, Ministry of Industry and Information Technology of the People’s Republic of China, Beijing, China

**Keywords:** 16S data, core microbiota, machine learning, meta - analysis, saliva microbiota

## Abstract

Saliva harbors a complex human microbiota closely linked to the occurrence and progression of various diseases. This meta-analysis of public 16S saliva data aimed to expand understanding of the microbiota’s associations with multiple diseases and explore its potential as molecular markers for multi-disease prediction, overcoming the limitation of single-disease-focused studies. From PubMed (2016–2024), 22 cohorts met the screening criteria (V3-V4 region 13 cohorts, V4 region 9 cohorts), comprising 7,750 samples. Bioinformatics analyses using QIIME2, Wekemo, and statistical modeling revealed saliva microbiota community characteristics, identified core microbes in the negative control group, and constructed a multi-disease prediction model based on 16S data. Key findings included: (1) significant differences in microbiota structure across physiological/pathological states (e.g., NPC groups resembled controls but diverged from colorectal cancer and PLHIV groups at the genus level); (2) Nine core microbiota, such as g:Streptococcus and g:Haemophilus_D_735815, were identified in the saliva samples of the negative control group; (3) robust classification performance of multi-class random forest models (AUC: 0.898–0.995 for V3-V4, 0.957–1 for V4). This study validated the feasibility of establishing healthy baselines via saliva microbiota and using machine learning for non-invasive disease diagnosis. Future research should expand disease coverage, increase sample sizes, and further investigate microbiota-disease associations to advance the development of non-invasive diagnostics.

## Introduction

Saliva harbors a highly complex human microbiota, whose compositional and functional alterations are closely linked to the onset and progression of a wide spectrum of oral and systemic diseases, including oral cancer, periodontitis, coronavirus disease 2019 (COVID-19), and non-oral malignancies such as colorectal cancer (Li et al., 2014; Sazanov et al., 2017). The clinical burden of these microbiota-associated diseases is substantial in China. According to the data in the “Cancer incidence and mortality in China, 2022” released by the National Cancer Center, the number of cases of oral cancer in 2022 will be 65,100, the crude incidence rate will be 2.76/100,000, and the number of deaths will be 35,200. The mortality rate is 1.33/100,000 (Zheng et al., 2024). According to the “January 2025 National Legal Infectious Disease Epidemic Overview” released by the Chinese Center for Disease Control and Prevention, coronavirus disease 2019 ranks fourth in the incidence of Class B infectious diseases, seriously endangering the health of the people (Prevention CCFDCA, 2025). Therefore, it is extremely necessary to study the relationship between salivary microbiota and diseases and explore their potential as diagnostic markers (Wong, 2012).

There have been several reports on salivary microbiota and human diseases. For example, Yu et al. studied the microbiota characteristics in the saliva of patients with oral lichen planus (OLP) in 2020 and compared them with recurrent aphthous ulcers (RAU). Compared, it was finally found that the microbiota in erosive OLP is significantly different from that in RAU, and changes in the microbiota may be related to the underlying disease process (Yu et al., 2020). Jiao et al. studied the changes of salivary microbiota in patients with thyroid cancer and thyroid nodules compared with healthy people in 2022 and finally determined that *g:Alloprevotella, g:Anaeroglobus*, etc. were significantly enriched in the thyroid cancer group (Jiao et al., 2022). In the 2024 study, Ren et al. used machine learning to explore the possibility of salivary microbiota becoming potential biomarkers of pulmonary nodules and only used these five bacterial characteristics to make the model perform better (AUC = 0.8) (Ren et al., 2024). However, most of these studies only focus on a single disease and its precursor lesions, demonstrating the impact of diseases on the composition of salivary microbiota from the perspective of diversity and species abundance, and lacking an overall assessment of human salivary microbiota. For the few studies that try to establish models, the sample size is usually small, especially the lack of healthy cohort data to increase the stability of the model, thus limiting the application of related results in disease diagnosis.

In this study, based on 16S public data of different diseases, we screened out 13 cohorts with sequencing regions V3-V4 and 9 cohorts with sequencing regions V4 from related studies from 2016 to 2024, totaling 7750 samples, and conducted a meta-analysis according to the sequencing regions. Based on this, we found the core microbiota of non-disease populations and tried to establish a multi-disease prediction model based on salivary microbiota. We hope to explore the connection between salivary microbiota and diseases from a multi-disease perspective through this study, explore the possibility of salivary microbiota as disease biomarkers, and the feasibility and practical significance of establishing a negative control baseline and training machine learning models to assist diagnosis.

## Materials and methods

### 2.1 Screening and preprocessing of salivary microbial 16S sequencing data

Considering the data volume and the level of technological development, We used the keyword “(Saliva) AND (16S)” to search the PubMed database for relevant literature (1114 articles in total) from 2016 to 2024. After that, we screened the retrieved literature. The screening conditions are as follows: (1) The sample data is 16S sequencing data of human saliva, and the sequencing region is V4 region or V3-V4 region (Teng et al., 2018; Zaura et al., 2021). (2) The total sample size of each study is not less than 40 cases. (3) Sequencing data is published and downloadable. (4) The metadata is complete. In the end, after removing disease groups with sample sizes of less than 20, we retained 13 cohorts with sequencing regions V3-V4 and 9 cohorts with sequencing regions V4, totaling 7750 cases, including 5231 samples in V3-V4 regions(3866 cases of control group and 1365 cases of disease group) and 2519 samples in V4 regions(1102 cases in control group and 1417 cases in disease group) (**Supplementary Figure 1**).

### 2.2 Bioinformatics analysis and visualization

Raw sequencing data were downloaded from the NCBI sra database (https://www.ncbi.nlm.nih.gov/sra) or the European Nucleotide Database (ENA) (https://www.ebi.ac.uk/). For 16S data, we divided them according to the sequencing regions and made manifest files respectively and then used QIIME2 (https://qiime2.org/) version 2023.9 to analyze the data. Firstly, the two sets of data were imported and integrated into qza files that can be processed by QIIME2, and then the original data were de-chimerized and denoised by DADA2 to generate amplicon sequence variant tables (ASVs). The generated results were used to calculate species dilution curves, and the representative sequences were used to construct phylogenetic trees for calculating species diversity. We assessed the relationship between disease and control groups by Bray-Curtis distance, subject separation based on pairwise distances, generated principal coordinate analysis (PCoA) plots using the first two principal coordinates and labeled according to disease group. We reflect the diversity of microbiota through the Evenness index and the Shannon index. We also performed species composition analysis based on BLAST (https://blast.ncbi.nlm.nih.gov/Blast.cgi) v2.13.0 method and machine learning method, compared the sequence similarity of species taxa and finally derived the species indexing feature table. The above analysis was implemented on the server using QIIME2 2023.9 and BLAST v2.13. 0 version. After that, we drew histograms of species abundance at the phylum level and genus level based on the derived abundance characteristic table.

We used linear discriminant analysis (LDA) effect size (LEfSe) (https://github.com/SegataLab/lefse) to analyze the differential microbiota of different disease groups of V3-V4 and V4 and the control group at the genus level. In addition, we analyzed the lefse results of different sequencing regions of the same sex in the control sample to explore the impact of different sequencing regions on the differential microbiota of men and women. In this analysis, the non-parametric Kruskal-Wallis rank sum was used to detect the species with significant abundance differences among different groups, and the groups with significant abundance differences were found. Then, linear discriminant analysis (LDA) was used to estimate the influence of each species’ abundance on the difference effect, and the linear regression scores of different species were obtained, and the species with significant differences (LDA > 3) were found. The above analysis was achieved by using the LEfSe mapping tool available at Wekemo Bioincloud (https://www.bioincloud.tech) using the abundance characteristic table (Yunyun Gao et al., 2024).

### 2.3 Statistics and modeling

We screened the control group microbiota abundance and inter-sample frequency of V3-V4 and V4 and extracted the microbiota with a frequency greater than 60% and a relative abundance greater than 1% in the sample, which was defined as the salivary core microbiota of the negative control group (Neu et al., 2021).

Next, we perform random forest training based on the results of the abundance feature table, randomly dividing 70% of the original data as the training set, and the remaining 30% as the test set. At the same time, in order to deal with the problem of unbalanced data volume, we undersample large sample groups, set 80% of the maximum sample size after undersampling as a threshold, and regard groups smaller than the threshold as small sample groups. Oversampling the training set of the small sample group by adaptive comprehensive sampling method (ADASYN), and maintaining the training set after oversampling to be no less than 80% of the maximum training set and no more than 90% of the maximum training set (Alhudhaif, 2021). After that, the first random forest multi-classification model training is performed on the pre-processed training set (the number of trees is set to 1000).And after the first training is completed, the number of features is re-selected according to the output cross_validation_plot.pdf file for secondary optimization training. In this secondary training process, 10-fold cross-validation is adopted to ensure the robustness of the model, and the number of trees is set to 500 to prevent over-fitting and under-fitting. Finally, for the optimized model, we extracted the top 20 features and plotted the ROC curves based on the One-vs-Rest (OvR) multi-classification strategy, to characterize the training performance of the optimized model.

## 3 Results

### 3.1 Data screening and quality control

We retrieved 1114 relevant articles using the PubMed database, excluded articles that did not meet the requirements according to the criteria described in Method 2.1, and obtained 22 cohorts, including 13 cohorts with sequencing regions V3-V4 and 9 cohorts with sequencing regions V4. Among them, there are 5,231 samples in V3-V4 regions, including 3,866 cases in the control group and 1,365 cases in the disease group. The size of 16S sequencing data of samples is 119.45 GB. There are a total of 2,519 samples in the V4 region, including 1,102 cases in the control group and 1,417 cases in the disease group. The 16S sequencing data size of samples is 15.86 GB. DADA2 quality control was performed on the data of V3-V4 regions, and the screening results of each group were as follows: 3553 samples in the control group (including 46.55% male and 50.04% female), 499 samples in the nasopharyngeal carcinoma (NPC) group, 71 samples in the Colorectal Cancer (CRC) group, and 58 samples in the Acquired Immunodeficiency Syndrome (PLHIV) group. After data preprocessing, the final sample size was 4,307, comprising 3,553 in the control group and 626 in the disease group. DADA2 quality control was performed on the data of V4 regions, and the screening results of each group were as follows: 1101 samples in the control group (including 51.86% male and 34.42% female), 770 samples in the coronavirus disease 2019 (SARS_CoV_2) group, 369 samples in the oral squamous cell carcinoma (OSSC) group, 128 cases in the epithelial precursor lesion (Pre-OSCC) group, 128 cases in the alcohol dependence(alcohol_dependence) group, and 22 cases in membranous nephropathy (MN) group. Before calculating the diversity, the feature table was filtered to remove those features that may be sequencing errors, noise, or low quality. Finally, the V3-V4 region participated in the diversity statistics of 3553 samples in the control group (including 46.55% male and 50.04% female), 499 samples in the NPC group, 69 samples in the CRC group, and 58 samples in the PLHIV group; In the V4 area, 1089 samples in the control group (52.43% male and 34.80% female), 748 samples in the SARS_CoV_2 group, 368 samples in the OSSC group, 128 samples in the Pre-OSCC group, 128 samples in the alcohol_dependence group and 22 samples in the MN group participated in the diversity statistics. In addition, the control group accounts for a relatively large proportion of samples (the V3-V4 region accounts for 82.49%, and the V4 region accounts for 43.72%), and the gender information is basically marked and distributed evenly. Therefore, we conducted a further analysis based on gender grouping in the control group after quality control of two sequencing regions.

### 3.2 Analysis of α diversity and β diversity of salivary microbiota

**Figure 1:**
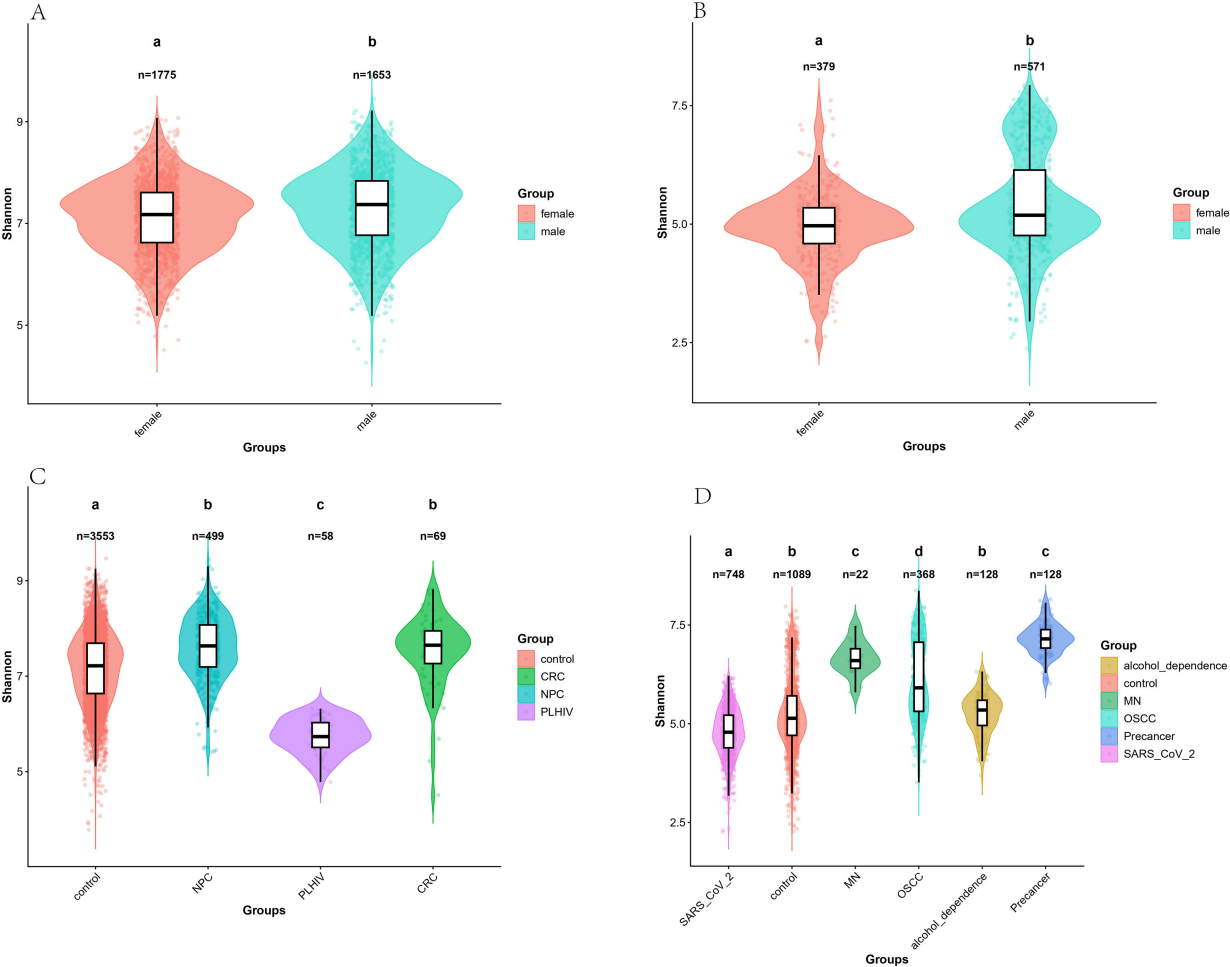
**(A)** Shannon index of V3-V4 region’s negative controls. **(B)** Shannon index of V4 region’s negative controls. **(C)** Shannon index of V3-V4 region. **(D)** Shannon index of V4 region. Different colors represent different sources. The different lowercase letters indicate statistically significant differences between groups (p < 0.05).

**Figure 2:**
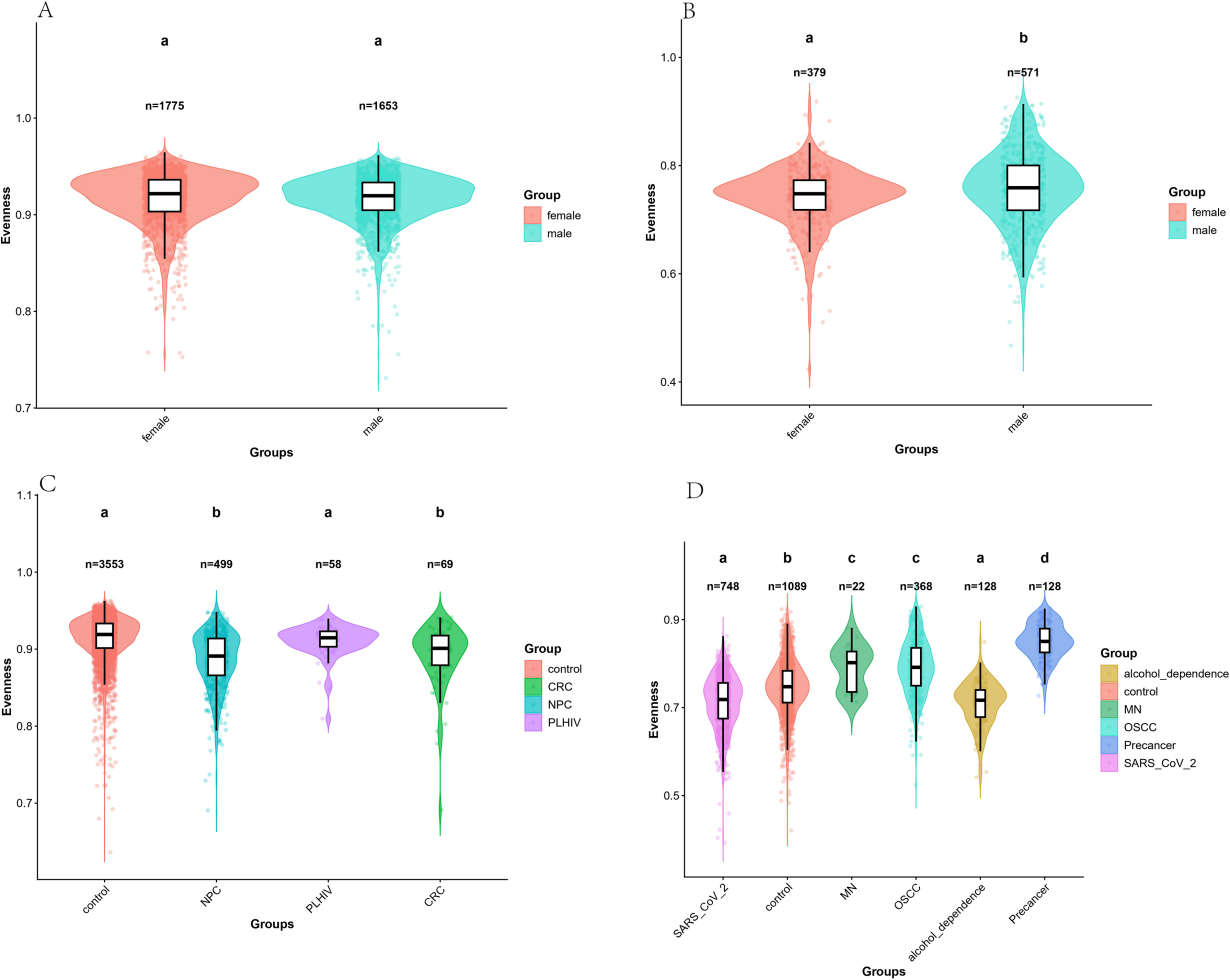
**(A)** evenness index of V3-V4 region’s negative controls. **(B)** evenness index of V4 region’s negative controls. **(C)** evenness index of V3-V4 region. **(D)** evenness index of V4 region. Different colors represent different sources. The different lowercase letters indicate statistically significant differences between groups (p < 0.05).

We analyzed the dilution curves for each group and observed that the curves of each group tended to be stable, indicating that the sampling depth of each group was sufficient for subsequent analysis (**Supplementary Figure 2**). Subsequently, we performed α diversity analysis on the screened data (**Figures 1**, **2**). We mainly analyze the Shannon and evenness indexes. Comparing the median data of each group, we found that the Shannon index among the four groups of data in the V3-V4 regions was the highest in the CRC group and the lowest in the PLHIV group. The others from high to low were the NPC group and the control group. The male group in the control samples was higher than the female group. Besides, there were significant differences between the control group and the CRC group, the NPC group and the PLHIV group (p < 0.05), there were significant differences between the PLHIV group and the CRC group and the NPC group (p<0.05), and there were significant differences between the male group and the female group in the control sample (p<0.05). As for the evenness index, the evenness index of the control group is the highest, the NPC group is the lowest, and the others are the PLHIV group and the CRC group from high to low. In the control samples, the female group is higher than the male group. There was a significant difference between the CRC group and the NPC group, the control group and PLHIV group (p < 0.05), there was no significant difference between the CRC group and the NPC group, the control group and the PLHIV group (p > 0.05), and there was no significant difference between male group and female group in the control sample (p > 0.05). In the six groups of data in the V4 region, the Shannon index was the highest in the Pre-OSCC group, the lowest in the SARS_CoV_2 group, and the others from high to low were the MN group, the OSCC group, the alcohol_dependence group, and control group. Among the control samples, the male group was higher than the female group. Besides, Significant differences were found between alcohol_dependence and the remaining five groups (p < 0.05). There were significant differences between the SARS_CoV_2 group and the OSCC group, MN group, and the Pre-OSCC group (p < 0.05). There was a significant difference between the OSCC group and the MN group, Pre-OSCC group (p < 0.05), and there was a significant difference between the male group and the female group in the control sample (p < 0.05). For the evenness index, the Pre-OSCC group was the highest, the alcohol_dependence group was the lowest, and others from high to low were the MN group, the OSCC group, the control group, and the SARS_CoV_2 group. In control samples, the male group was higher than the female group. There were significant differences between the control group and each disease group (p < 0.05), and Significant differences were observed between the SARS_CoV_2 group and the other four disease groups (p < 0.05). There was a significant difference between the Pre-OSCC group and the OSCC group and the MN group (p < 0.05), and there was a significant difference between the male group and the female group in the control sample (p < 0.05).

We performed a dimensionality reduction analysis on the screened data through the PCoA method. From the results of the PCoA analysis (**Supplementary Figure 3**), for the V3-V4 region, there is a complete separation between the four groups. Most sample points in the disease group do not overlap with those in the control group. At the same time, the distribution of sample points in the control group is more scattered than those in the disease group, indicating that the samples within the group are more different. The contribution degrees of PCo1 and PCo2 in the four groups of data in V3-V4 regions were low (12.31% for PCo1 and 6.561% for PCo2). For the V4 region, the control group, alcohol_dependence group, and the OSCC group overlapped to a certain extent, and the MN group and Pre-OSCC group also overlapped to a certain extent, while the Pre-OSCC group was completely surrounded by the OSCC group. The SARS_CoV_2 group was further away from the other groups, and the sample points were more concentrated, with only a small overlap with the alcohol_dependence group. The contribution values of PCo1 and PCo2 were higher in the six sets of data (23.15% for PCo1 and 19.34% for PCo2). However, for control samples, the male and female groups in V3-V4 and V4 regions overlap greatly, and the contribution of PCo1 and PCo2 is generally low (12.95% for PCo1 and 4.508% for PCo2 in the V3-V4 regions, 32.41% for PCo1 and 8.604% for PCo2 in the V4 regions).

### 3.3 Species composition of salivary microbiota and identification of core microbiota of control group

**Figure 3:**
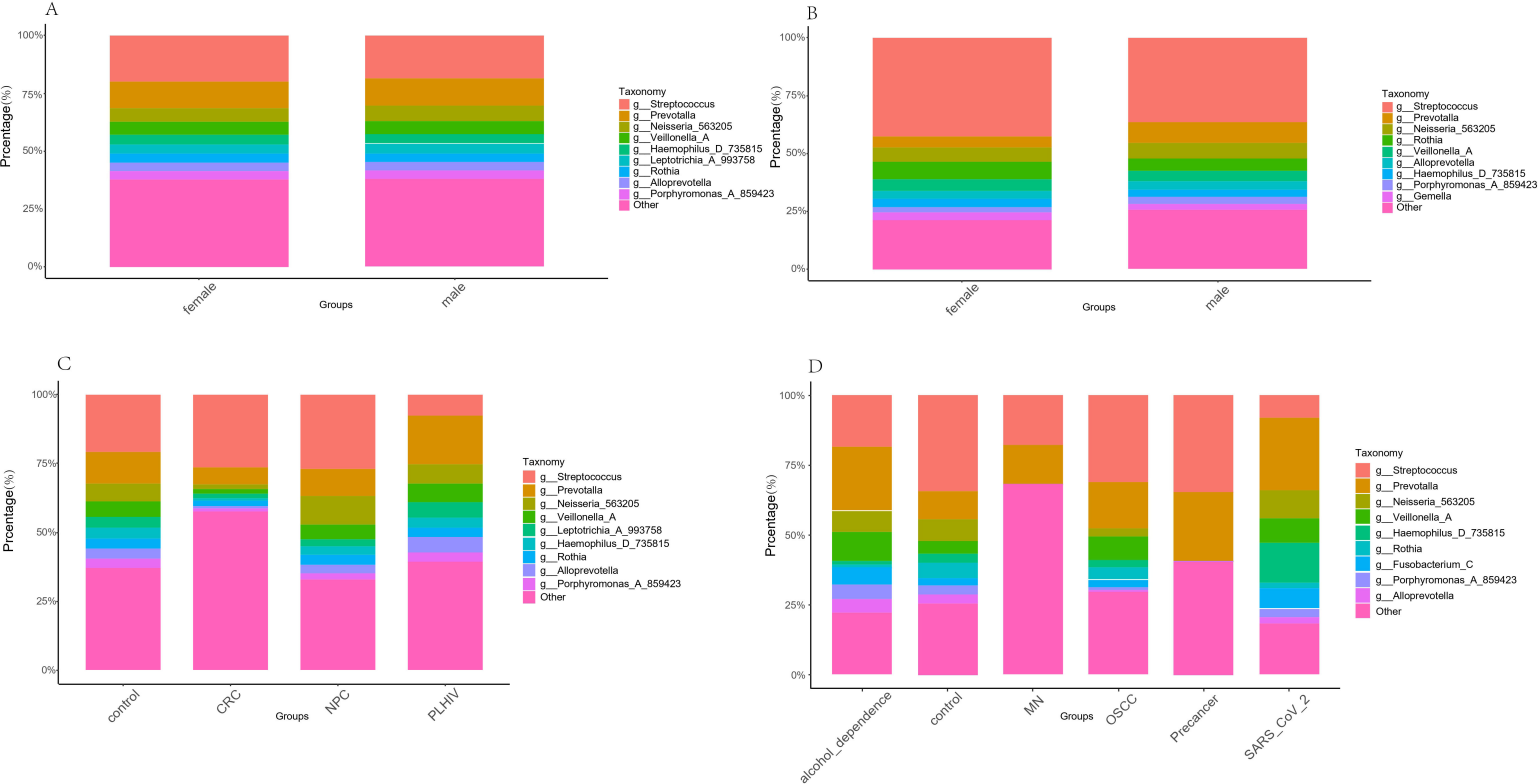
**(A)** Species composition at the genus level in V3-V4 region’s negative controls. **(B)** Species composition at the genus level in V4 region’s negative controls. **(C)** Species composition at the genus level in V3-V4 region. **(D)** Species composition at the genus level in V4 region. Different colors represent different bacteria.

To analyze the composition of salivary microbiota, we plotted the percentage bar charts of salivary microbial microbiota in each group at the phylum level and genus level according to the sequencing regions V3-V4 and V4, respectively. The phylum-level microbiota composition analysis (**Supplementary Figure 4**) based on the V3-V4 region shows that the dominant microbiota in the control group, CRC group, and NPC group is predominantly p:Firmicutes_D. The specific distribution characteristics are as follows: control group: the dominant microbiota is p*:*Firmicutes_D, p:Bacteroidota and p:Proteobacteria, and the characteristics of the male group and the female group in the control sample are consistent with them. In the CRC group, p:Firmicutes_A replaced p:Proteobacteria as the second dominant microbiota, and the top three were p:Firmicutes_D, p:Firmicutes_A and p:Bacteroidota. The top three NPC groups account for the same proportion as the control group. Although there are differences in the proportion among the three groups, the overall community structure at the phylum level is highly conservative, and the composition of the dominant microbiota is not much different. However, the PLHIV groups’ top three accounted for p:Bacteroidota, p:Firmicutes_A, and p:Proteobacteria, which were quite different from the dominant microbiota groups in the first three groups. The analysis of microbiota composition at the genus level (**Figure 3**) showed that *g:Streptococcus*, *g:Prevotella*, and *g:Neisseria_563205* were the dominant genera in the control group, and the proportion of the three genera decreased stepwise. The characteristics of the male group and female group in the control sample were consistent with them. The top two bacterial genera in the CRC group were *g:Streptococcus* and *g:Prevotella*, respectively, and other bacterial genera (including unnamed groups) accounted for lower proportions. NPC group: The composition of the dominant bacteria is consistent with that of the control group, but the difference in the proportion of *g:Neisseria_563205* and *g:Prevotella* is narrower. The PLHIV group showed a unique distribution pattern, with *g:Prevotella* as the single dominant bacterial genus, while *g:Streptococcus*, *g:Neisseria_563205*, *g:Veillonella_A*, and *g:Leptotrichia_A_993758* accounted for similar proportions. At the genus level, the control group was close to the NPC group, but different from the CRC and PLHIV groups. The microbiota composition analysis of the V4 phylum level showed that the dominant microbiota of the control group were p:Firmicutes_D, p:Proteobacteria, and p:Bacteroidota, which were close to the results of V3-V4 sequencing regions, and the characteristics of male and female groups in control samples were consistent with them. In the alcohol_dependence group, p:Bacteroidota was the most dominant microbiota, followed by p:Firmicutes_D, while p:Proteobacteria and p:Firmicutes_C accounted for similar proportions. In the MN group, the top three microbiota, p:Bacteroidota, p:Firmicutes_D, and p:Proteobacteria, were dominant. The top three dominant microbiota in the OSCC group are p:Firmicutes_D, p:Bacteroidota, and p:Proteobacteria, which are similar to those in the control group. The top three dominant microbiota in the Pre-OSCC group were p:Firmicutes_D, p:Bacteroidota, and p:Actinobacteriota. The SARS_CoV_2 group showed a unique distribution, with p:Bacteroidota and p:Proteobacteria accounting for similar proportions and ranking first, followed by p:Fusobacteriota, while p:Firmicutes_D and p:Firmicutes_C accounting for little difference. The analysis of microbiota composition at the genus level in the V4 region showed that there were significant differences in microbiota structure among different cohorts. In the control group, *g:Streptococcus*, *g:Prevotella*, and *g:Neisseria_563205* constituted the top three dominant microbiota, and the characteristics of the male group and female group in the control sample were consistent with them; The microbiota structure of the alcohol_dependence group changed significantly, and *g:Prevotella* jumped to the first place, followed by *g:Streptococcus* and *g:Veillonella_A*. It is worth noting that the MN group and the Pre-OSCC group showed unique common characteristics: *g:Streptococcus* and *g:Prevotella* both showed a high proportion and similar proportion, forming the common characteristics of dual dominant bacteria. The OSCC group showed significant enrichment of *g:Streptococcus*, and its proportion was significantly higher than that of the subsequent *g:Prevotella* and *g:Veillonella_A*. The distribution of bacterial microbiota in the SARS_CoV_2 infected group was also specific, and *g:Prevotella*, *g:Haemophilus_D_735815*, and *g:Neisseria_563205* constituted the top three dominant bacterial genera.

We identified the core microbiota of salivary microbiota in the control group of V3-V4 regions and V4 regions (**Supplementary Figure 5**) according to the method2.2 and finally found that *g:Streptococcus*, *g:Haemophilus_D_735815*, *g:Prevotella*, *g:Granulicatella*, *g:Veillonella_A*, *g:Neisseria_563205*, *g:Gemella, g:Porphyromonas_A_859423, g:Rothia* are common microbiota of two sequencing regions.

### 3.4 Identification of abundance difference of salivary microbiota

**Figure 4:**
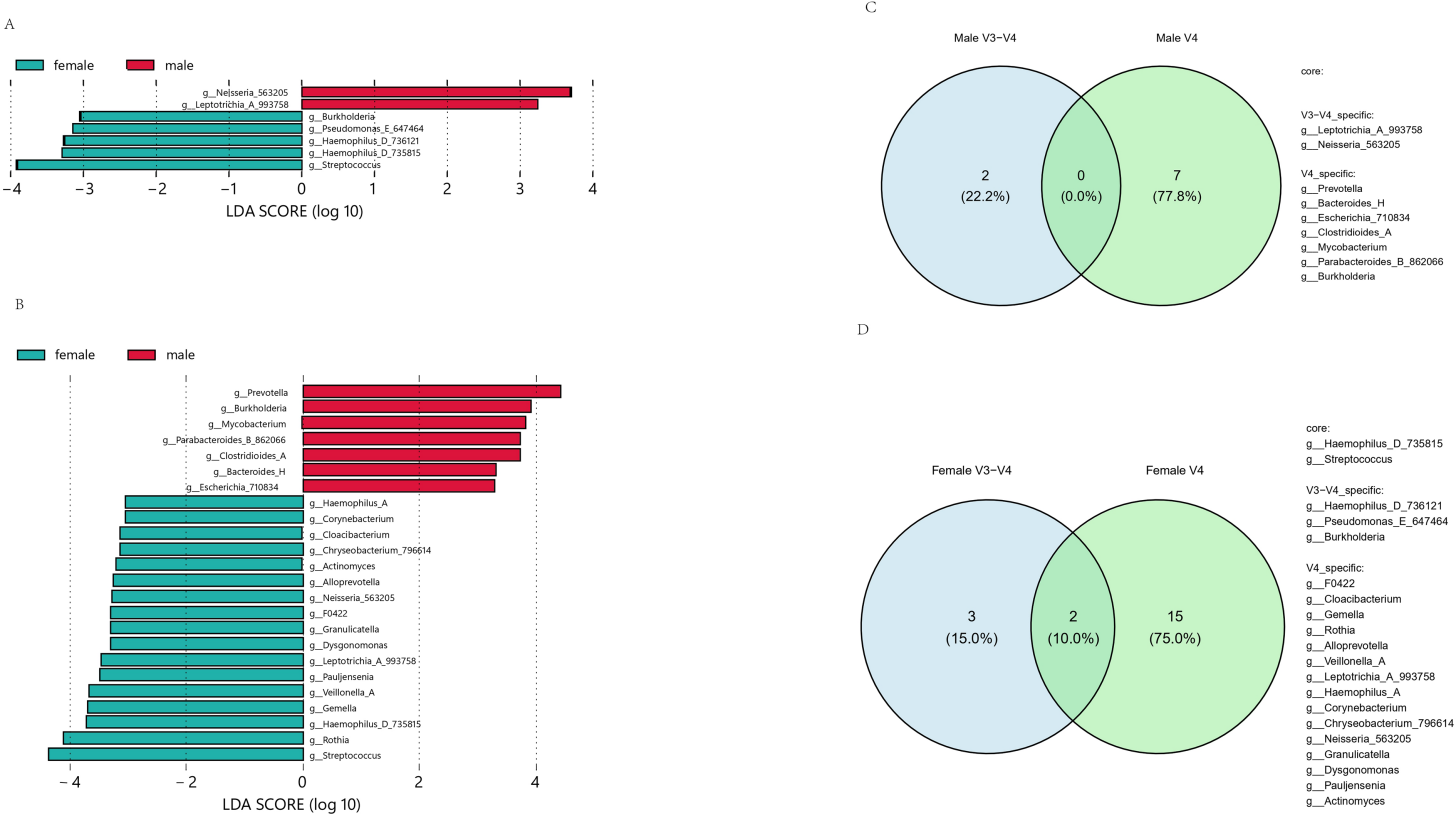
**(A)** LEfSe of V3-V4 region’s negative controls. **(B)** LEfSe of V4 region’s negative controls. **(C)** Venn diagram of male negative controls’ differential microbiota. **(D)** Venn diagram of female negative controls’ differential microbiota. Different colors represent different sources.

In order to explore the difference in abundance level between the microbial samples of the disease group and the control group, we conducted abundance differential microbiota analysis in V3-V4 and V4 regions respectively. According to the analysis results (**Figure 4**; **Supplementary Figure 6**), the number of bacteria with differences in the relative abundance of salivary microbiota in the CRC group, NPC group, PLHIV group, and control group in the V3-V4 region is 28 genera, 5 genera, 11 genera, and 14 genera, respectively. Among them, *g_Streptococcus*, *g_Nanosyncoccus*, g_CAG_273 and *g_Oribacterium* were the most different in the CRC group (LDA Score > 4.0); *g_Neisseria_563205* showed the greatest difference in the NPC group (LDA Score > 4.0); *g_Prevotella*, *g_Alloprevotella*, *g_Veillonella_A*, *g_Leptotrichia_A_993758* were the largest differences in the PLHIV group (LDA Score > 4.0); In the control group of V3-V4 regions, *g_Haemophilus_D_735815* and *g_Porphyromonas_A_859423* had the largest difference (LDA Score > 4.0). In addition, there were 7 groups of bacterial genera differences between the male group and female group in the control sample. The number of microbiota with differences in the relative abundance of salivary microbiota in the MN group, SARS_CoV_2 group, alcohol_dependence group, OSCC group, and control group in the V4 region was 10 genera, 9 genera, 15 genera, 17 genera, 5 genera, and 12 genera, respectively. Among them, *g:Bacteroides_H, g:Parabacteroides_B_862066, g:Escherichia_710834, g:Staphylococcus* differed the most in the MN group (LDA Score > 4.0); *g_Burkholderia, g_Mycobacterium, g_Clostridioides_A* differences were greatest in the Pre-OSCC group (LDA Score > 4.0); g*_Prevotella, g_Haemophilus_D_735815, g_Neisseria_563205, g_Fusobacterium_C, g_Leptotrichia_A_993758* had the greatest differences in the SARS_CoV_2 group (LDA Score > 4.0); *g_Veillonella_A*, *g_Porphyromonas_A_859423, g_Alloprevotella*, *g_Porphyromonas_A_859424* differences were greatest in the alcohol_dependence group (LDA Score > 4.0), and Pauljensenia differences were greatest in the OSCC group (LDA Score > 3.0); In the last V4 region, *g_Streptococcus*, *g_Rothia and g_Gemella* showed the largest difference in control group (LDA Score > 4.0). In addition, there were 24 groups of bacterial genera differences between the male group and female group in the control sample.

In order to explore whether the sequencing region will affect the identification of differential microbiota in the control group of different genders, we analyzed the salivary microbiota of the control group of different sequencing regions of the same gender based on the above lefse results. The results showed that there were fewer differential microbiota. In the control sample, the male group detected 0% of the common microbiota in both sequencing regions at the same time, and the female group detected 10% of the common microbiota in both sequencing regions at the same time.

### 3.5 Multi-disease prediction model based on salivary microbial 16S data

**Figure 5:**
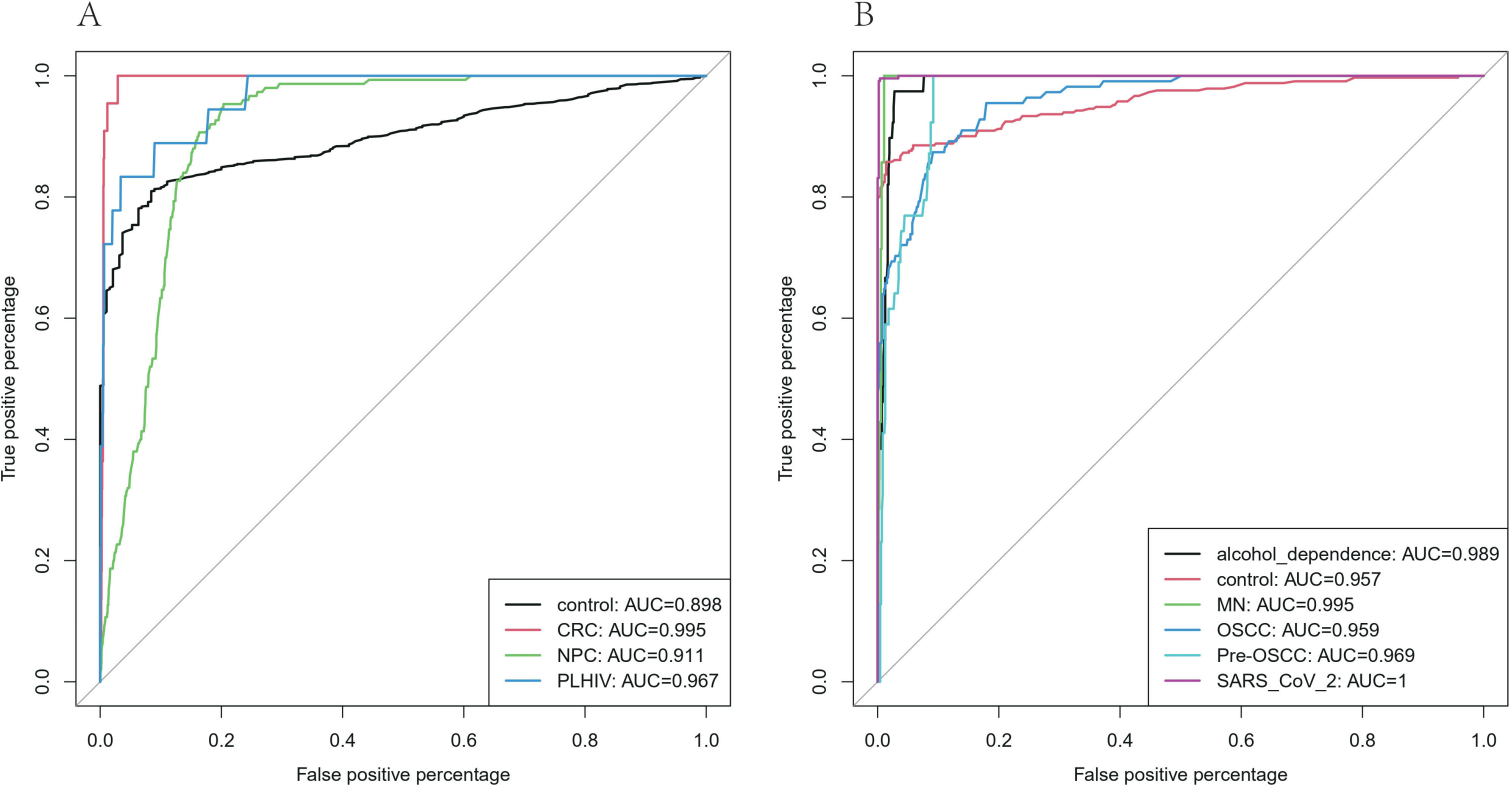
**(A)** ROC curve of V3-V4 region. **(B)** ROC curve of V4 region. Different colors represent different sources.

**Figure 6:**
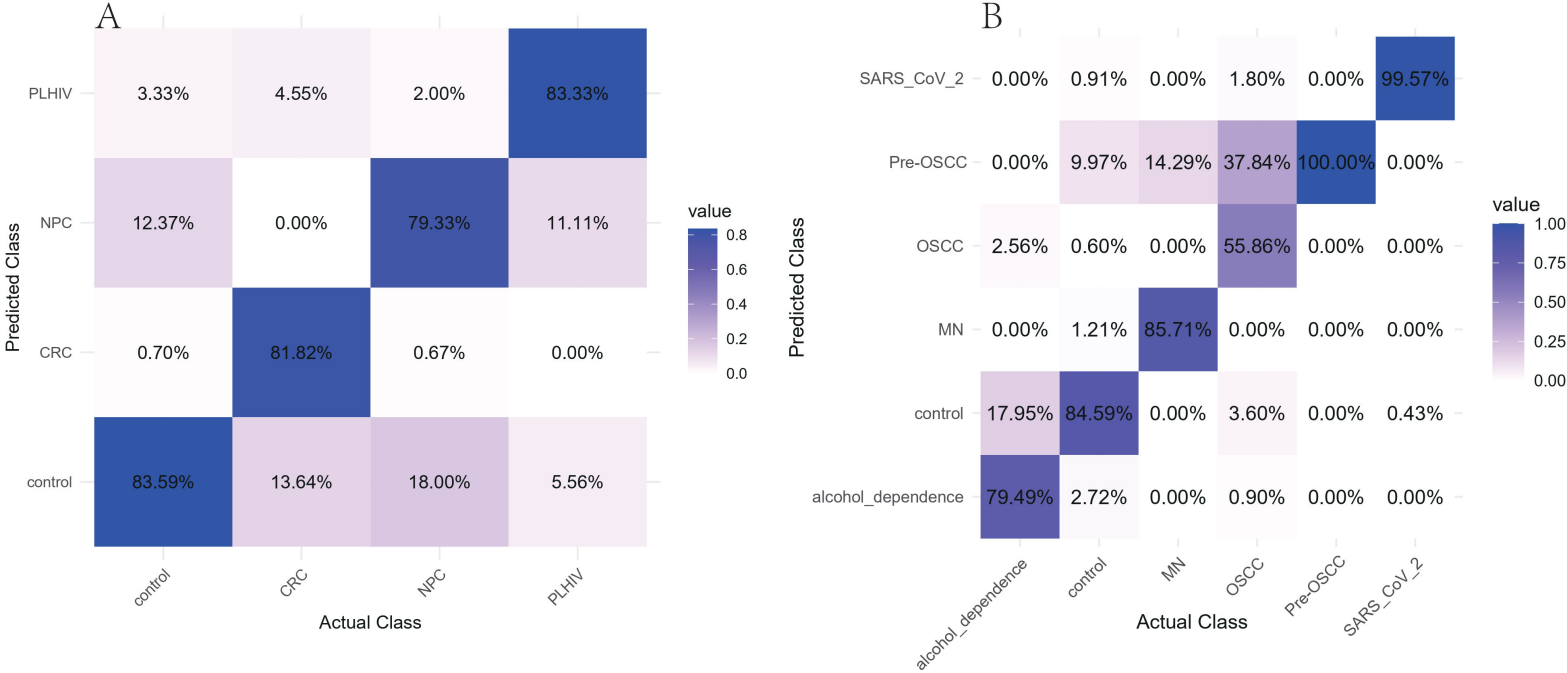
**(A)** Test set confusion matrix of V3-V4 region. **(B)** Test set confusion matrix of V4 region. Different colors represent different sources.

Based on the 16S data of oral saliva, in this study, we respectively trained multi-class random forest models of V3-V4 regions and V4 regions. For the test results of the V3-V4 regions, the AUC values (**Figure 5**) of each group ranged from 0.898 to 0.995, with the highest being 0.995 in the CRC group and the lowest being 0.898 in the control group. The test accuracy (**Figure 6**) in the confounding matrix was 83.59% in the control group, 81.82% in the CRC group, 79.33% in the NPC group, and 83.33% in the PLHIV group. Among the top 20 features (**Supplementary Figure 7**), the Mean Decrease Accuracy (MDA) values were all greater than 9, with the highest value observed in *g:Prevotella*. For the test results of the V4 region, the AUC values of each group ranged from 0.957 to 1, with the highest being 1 in the SARS_CoV_2 group and the lowest being 0.957 in the control group. The test accuracy of alcohol_dependence was 79.49%, control was 84.59%, MN was 85.71%, OSCC was only 55.86%, and 37.84% were classified as Pre-OSCC, Pre-OSCC was 100%, SARS_CoV_2 was 99.57%.Among the top 20 features, the MDA values were all greater than 13.5, with the highest value observed in *g:Streptococcus*.

## 4 Discussion

As a human organ rich in microbiota, the regulation of the interaction between the microbiota and the host in the oral cavity affects the key aspects of the normal physiology, metabolism, immunity, and neurological functions of the host (Xiao et al., 2020). Therefore, saliva is a biomarker used to diagnose, predict, and monitor diseases, and has the advantage of easy access. However, most current studies only focus on a single disease, demonstrating the impact of diseases on the composition of salivary microbiota from the perspective of diversity and species abundance, and lacking an overall understanding of salivary microbiota. Although some studies have paid attention to the influence of gender and other factors on the salivary microbiota of negative controls, they lack the identification of the core microbiota of the control group. For example, before this study, Wang et al. had tried to use saliva-related 16S sequencing data in the 2023 study to conduct auxiliary diagnosis research on metabolic-associated fatty liver (MAFLD) disease through random forest machine learning methods. Although the training results showed a good classification accuracy rate (AUC is 0.82), but only a single disease connection was established and the amount of data was small (10 people in the control group and 10 people in the case group), lacking a large cohort to increase the stability of the model (Wang et al., 2023). For studies on healthy large cohorts, such as Odendaal et al. ‘s study in 2024, although it has established connections with some seasonal diseases, it covers three sampling location data of saliva, oropharynx, and nasopharynx, and lacks unified markers to define healthy people (Odendaal et al., 2024). Willis et al. studied the saliva 16S data of healthy people in 2018 but lacked a connection with disease (Willis et al., 2018). To break through these limitations, this study selected the 16S data of salivary microbiota in V3-V4 and V4 regions, which are rich in public data, to carry out a meta-analysis, combined the same disease data in different cohorts, built a baseline of salivary microbiota in negative controls, and constructed a multi-disease classification machine learning early warning model based on saliva for the first time. This study aims to expand the cognition of the relationship between salivary microbiota and diseases and explore the potential value of salivary microbiota as molecular markers of diseases.

We analyzed the species diversity of the V3-V4 and V4 regions based on 16S data. We used Shannon and evenness indices to characterize alpha diversity. The evenness and Shannon index will be calculated, representing the evenness and richness of the community respectively. We observed that the Shannon index of the control group was lower than that of the CRC and NPC groups and higher than that of the PLHIV group for the V3-V4 regions, and the difference between the three groups was statistically significant (p < 0.05). The evenness index was higher than that of the three disease groups, but only the difference between CRC and NPC was statistically significant (p < 0.05). In fact, Ma et al. pointed out in their 2023 study that the decrease of symbiotic species and the increase of pathogenic taxa can be observed in PLHIV (Ma et al., 2023). Zhang et al. pointed out in their 2016 study that the species of microbiota in the saliva of oral tumor patients’ Diversity and relative abundance are greater than those of healthy people (Zhang et al., 2016). The Shannon index of the control group in the V4 region was only higher than that of the SARS_CoV_2 group but lower than that of alcohol_dependence, MN, OSCC, and Pre-OSCC groups, and there was no statistically significant difference with alcohol_dependence only (p > 0.05). The evenness index was higher than alcohol_dependency, SARS_CoV_2, and lower than MN, OSCC, and Pre-OSCC, while there was a statistically significant difference for all disease groups (p < 0.05). We observed a general elevation in α-diversity indices (including the Shannon and Evenness indices) in the disease groups for both the V3-V4 and V4 hypervariable regions. This shift in α-diversity under disease conditions is consistent with previous findings that oral microbial community structure is closely associated with disease status and host immune function, which is consistent with the research results of Zhang et al. in 2019 (Zhang et al., 2019). For SARS_CoV_2, Gupta et al. also pointed out in the 2022 study that the α diversity of patients decreased (Gupta et al., 2022). Furthermore, the male group in the control samples of both sequenced regions was higher than the female group in the Shannon index, which is consistent with the results of Vinerbi, E. et al. in the study in 2024 (Vinerbi et al., 2024).

We performed a species composition analysis at the genus level for data from regions V3-V4 and V4. For each disease group, we found a high proportion of *g:Streptococcus* within CRC in the V3-V4 region, while Qu et al., 2023 found that endocarditis and bacteremia were associated with Streptococcus. gallolyticus infection increases the risk of CRC and exposure to Streptococcus. gallolyticus antigen is strongly associated with an increased incidence of CRC (Qu et al., 2023). The relative abundance of *g:Neisseria_563205* was the highest in the NPC group, and Zhang et al. found the same result in a 2015 study (Zhang et al., 2015). The proportion of *g:Prevotella* was higher in the PLHIV group, and Nannini et al. found the same results in a 2024 study (Nannini et al., 2024). For the high proportion of *g:Bacteroides_H* in the MN group in the V4 region, although there are few studies on the relationship between MN and salivary microbiota, Zafar et al. pointed out in their 2021 study that *g:Bacteroides*, a probiotic intestinal microbiota, can transfer virulence genes when it appears outside the intestine, thereby providing sister cells and adjacent cells with virulence factors that may contribute to the pathogenesis of extraintestinal organs (Zafar and Saier, 2021). The proportion of the *g:Prevotella* genus is relatively high within the SARS_CoV_2 group, while Gupta et al. pointed out in their 2022 study that the lung microbiota is more related to the microbiota of the oropharynx than that of the nasopharynx and gastrointestinal tract. At the same time, *g:Prevotella* is overrepresented in SARS_CoV_2 patients, so it may be involved in the production of proteins that can promote SARS_CoV_2 infection and disease severity (Gupta et al., 2022). The proportion of *g:Veillonella* in the alcohol_dependence group is relatively high. Although alcohol_dependence studies tend to focus on neurological or genetic mechanisms, changes in microbial composition are consistent with Lang et al. ‘s stool test results of patients with alcoholic hepatitis in 2020. Consistent results may indicate that alcohol has a consistent effect on microbiota in the digestive tract (Lang et al., 2020). Pre-OSCC is a precursor disease of OSCC. We focused on the comparative analysis of the two and found that there are differences in *g:Fusobacterium*, *g:Prevotella*, *g:Haemophilus*, and other parts in OSCC and Pre-OSCC. Saikia et al. also believed that these microbiotas play a role in the definition of cancer staging in the study in 2023 (Saikia et al., 2023). In addition, compared with the results of Liu et al. ‘s metagenomic study in 2022, the result of the higher proportion of *g:Streptococcu* in the female group in the control sample is consistent, but the results of *g:Prevotella* and *g:Veillonella* have a higher proportion in the opposite sex (Liu et al., 2023). Combined with the analysis of differential microbiota in different sequencing regions of the same sex, there may be no specific differential microbiota in the negative control, and the results are greatly affected by the selection of sequencing regions. This phenomenon can be mainly attributed to the inherent bias of the two hypervariable region amplification primers (Sunthornthummas et al., 2025).The top 20 features ranked by Mean Decrease Accuracy (MDA) were generally consistent with the differential bacterial genera identified by LEfSe analysis, which verified the rationality of the discriminant method for “disease-associated characteristic microbiota”. However, several exceptions were observed: for instance, *g:Fusobacterium_A* and *g:Mesorhizobium_F_498388* in the V3-V4 region, as well as *g:Faecousia* in the V4 region, were included in the MDA top 20 features but not identified as differential taxa by LEfSe. Meanwhile, we noted that the unclassified genus exhibited a high MDA score in the V3-V4 region, which may be attributed to the inherent limitations of 16S rRNA gene sequencing in taxonomic identification and the constraints of traditional methods for characteristic microbiota identification.

We identified the core microbiota of the control group in the V3-V4 and V4 regions of saliva. A total of nine common microbiota were consistently detected across both sequencing regions: *g:Streptococcus*, *g:Prevotella, g:Veillonella_A*, *g:Neisseria_563205*, *g:Porphyromonas_A_859423*, and *g:Rothia*. These can be considered as the core microbiota of negative controls. The first four genera align closely with the common salivary microbiota reported by Segata et al. in 2012. Furthermore, this study highlights that *g:Streptococcus* is frequently observed in the buccal mucosa, keratinized gingiva, and hard palate, while *g:Rothia* and *g:Porphyromonas* are also prevalent in feces, suggesting a degree of consistency in the microbiota composition across the digestive system (Segata et al., 2012). Additionally, specific common microbiota unique to the V3-V4 region were identified, including *g:Fusobacterium_C*, *g:Leptotrichia_A_993758*, *g:Pauljensenia*, *g:Alloprevotella*, and *g:Actinomyces*. No such specific microbiota were detected in the V4 region alone, potentially due to the difference of sequencing and longer sequencing interval of the V3-V4 region, which may enhance the accuracy of results. It is worth noting the potential reference value of these specific common microbiota in the V3-V4 region for defining the core microbiota of negative controls (Fadeev et al., 2021).

We used the integrated saliva 16S data to conduct machine learning training in the random forest. From the test results of the test set, the AUC values of the V3-V4 group were all higher than 0.898, and the AUC values of the V4 group were all higher than 0.957, showing high classification performance and anti-interference ability while being less affected by the threshold. As a result of this pair of confusion matrices, the correct classification rate of V3-V4 regions is between 79.33% and 83.59%, with the highest being 83.59% in the control group and the lowest being 79.33% in the NPC group. At the same time, we noticed that the disease group test set Most of the errors are concentrated in being classified as the control group, which may be due to the unbalanced proportion of original data in the V3-V4 region (76.10% after undersampling the control group) caused by category imbalance bias. Except for OSCC, whose correct rate of was 55.86%, the correct rate of other groups in the V4 region was above 79.49%, which proved the feasibility of salivary microbiota 16S data-assisted diagnosis. At the same time, 37.84% of the OSCC group were classified as Pre-OSCC, which may indicate that as a precursor lesion of OSCC, the microbial composition of pre-OSCC and OSCC has a certain similarity, and there may be significant changes in the salivary microbial composition in the early stage of the whole process which affects the development of the disease (Lin et al., 2021). This point is consistent with the PCoA results described above. In addition, the 100% accuracy rate of Pre-OSCC may also be due to the random error caused by the small test sample (39 cases in the test set). After we combined Pre-OSCC and OSCC weighted into one class, the prediction accuracy reached 95.33%.Although it is not possible to achieve 100% classification accuracy, this is the normal phenomenon of classification models by machine learning. The small number of disease samples misclassified in this study were within an acceptable and reasonable error range and did not affect the overall excellent performance of the model and the core research conclusions.

However, this study has several limitations. First, there was non-negligible heterogeneity among the included cohorts. We systematically collated the technical details and baseline population characteristics of all the included cohorts in **Supplementary Table 1**, including the between-cohort differences in key dimensions: sample extraction methods, sequencing platforms, geographic distribution of the study population, and sex composition. This technique and population-level heterogeneity may have a potential impact on the diagnostic performance of the model. At the same time, because all the data used in this study were obtained from public online databases, there were substantial missing values for several key clinical covariates (e.g., smoking history), which limited our ability to perform deeper stratified analyses and statistical adjustments for some sources of heterogeneity. Therefore, based on the data quality and the core scientific questions of this study, we conducted targeted analysis and discussion on the two key heterogeneity factors of sequencing region and gender. Secondly, due to the limitation of public data accessibility, the types of included diseases and the number of cases of each disease show obvious unbalanced distribution, which may have a certain impact on the training performance and generalization ability of the random forest model. In addition, all sequencing data included in this study were generated by amplicon sequencing technology. As a result, most species-level ampliconoid sequence variants (ASVs) are annotated only to the genus level, which prevents more refined species-level annotation and functional analysis. Despite the above limitations, the existing results of this study initially established a systematic and overall understanding of the association between salivary microbiota and various diseases, and the constructed multi-class disease diagnostic model also showed good reliability. In the future studies, we will further explore the biological mechanisms of these associations through wet-laboratory experiments, expand the disease spectrum and sample size, control the interference of heterogeneity between cohorts through standardized experimental design and multi-dimensional hierarchical analysis, and further reveal the potential value of salivary microbiota as a non-invasive biomarker of diseases.

## 5 Conclusion

In this study, we systematically reviewed 13 cohorts focusing on the V3-V4 region and 9 cohorts on the V4 region of the 16S sequencing data, encompassing a total of 7,750 samples. Our findings were preliminarily validated through machine learning. Regarding the microbial composition, we identified nine core bacterial genera in healthy individuals, including g:Streptococcus, g:Haemophilus_D_735815, and g:Prevotella etc. The optimized machine learning model demonstrated excellent classification performance on the validation dataset, underscoring the feasibility of establishing a microbial baseline for health assessment and developing non-invasive diagnostic tools using salivary microbiota. These results highlight the potential of salivary microbiome analysis as a novel approach to disease diagnosis.

## Data Availability

The original contributions presented in the study are included in the article/**Supplementary Material**. Further inquiries can be directed to the corresponding authors.

## Supplementary material

Supplementary Figure 1 cohorts screening workflow.n represents the number of cohorts.

Supplementary Figure 2 **(A)** species dilution curves of V3-V4 region’s negative controls. **(B)** species dilution curves of V4 region’s negative controls. **(C)** species dilution curves of V3-V4 region. **(D)** species dilution curves of V4 region. Different colors represent different sources.

Supplementary Figure 3 **(A)** PCoA analysis of V3-V4 region’s negative controls. **(B)** PCoA analysis of V4 region’s negative controls. **(C)** PCoA analysis of V3-V4 region. **(D)** PCoA analysis of V4 region. Different colors represent different sources, Each point represents a sample.

Supplementary Figure 4 **(A)** Species composition at the phylum level in V3-V4 region’s negative controls. **(B)** Species composition at the phylum level in V4 region’s negative controls. **(C)** Species composition at the phylum level in V3-V4 region. **(D)** Species composition at the phylum level in V4 region. Different colors represent different bacteria.

Supplementary Figure 5 Venn diagram of core microbiota.

Supplementary Figure 6 **(A)** LEfSe of V3-V4 region. **(B)** LEfSe of V4 region. Different colors represent different bacteria.

Supplementary Figure 7 **(A)** Top 20 feature of V3-V4 region. **(B)** Top 20 feature of V3-V4 region.

Supplementary Table 1 Total sample metadata.

Supplementary Table 2 negative controls’ metadata.
