# Supplementary material for: Salivary microbial meta-analysis reveals gender differences in oral microbiota, core microbiota, and molecular markers
Source: Front Cell Infect Microbiol. 2026 Apr 15;16:1796284. doi: 10.3389/fcimb.2026.1796284 (PMC13125147; doi:10.3389/fcimb.2026.1796284)
Supplement: Supplementary Figure 1 — cohorts screening workflow.n represents the number of cohorts. [file DataSheet1.pdf]

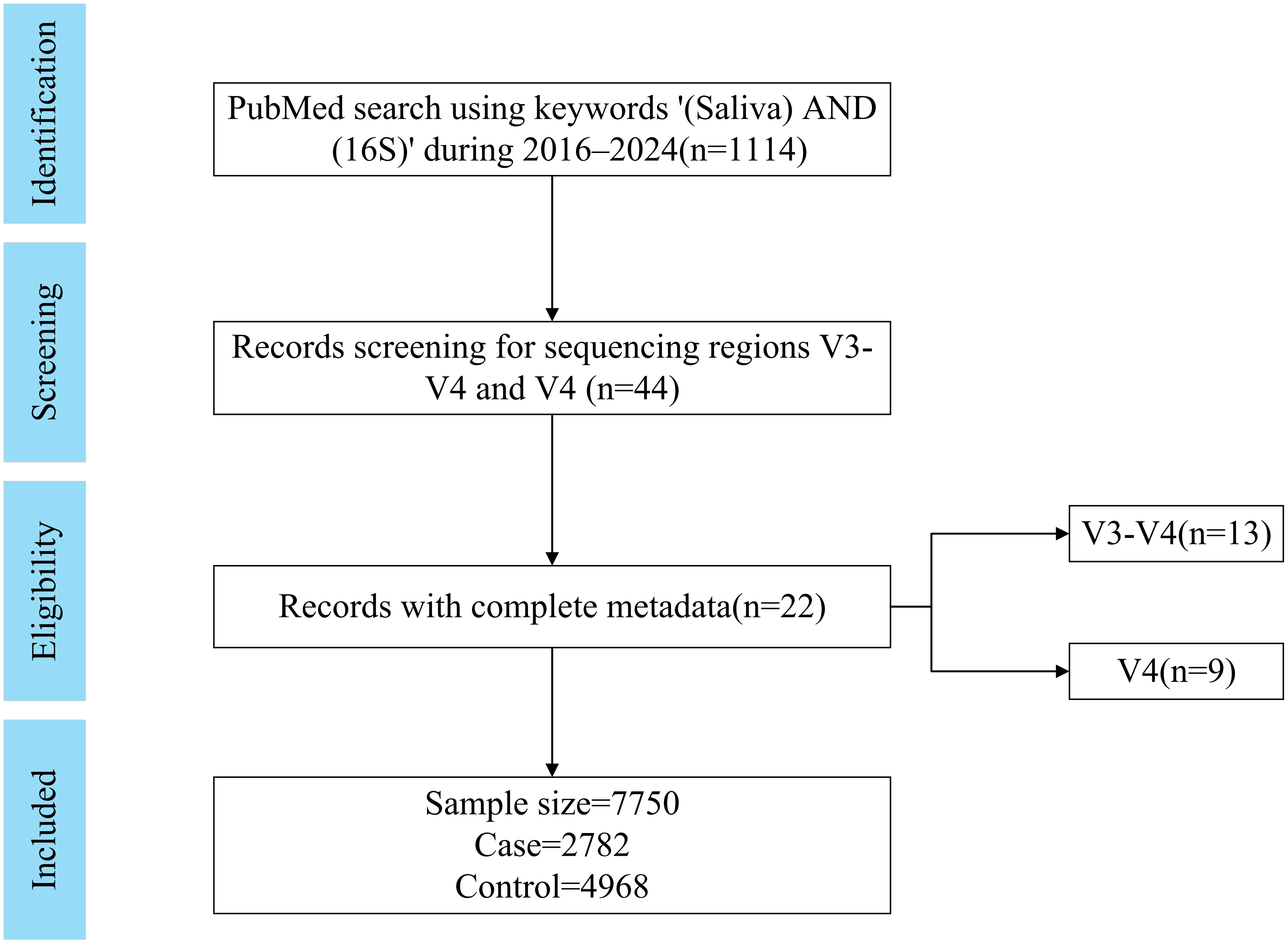
Identification
Screening
Eligibility
Included
PubMed search using keywords '(Saliva) AND (16S)' during 2016–2024(n=1114)
Records screening for sequencing regions V3-V4 and V4 (n=44)
Records with complete metadata(n=22)
V3-V4(n=13)
V4(n=9)
Sample size=7750
Case=2782
Control=4968
