# Supplementary figures and images for: Salivary microbial meta-analysis reveals gender differences in oral microbiota, core microbiota, and molecular markers
Source: Front Cell Infect Microbiol. 2026 Apr 15;16:1796284. doi: 10.3389/fcimb.2026.1796284 (PMC13125147; doi:10.3389/fcimb.2026.1796284)

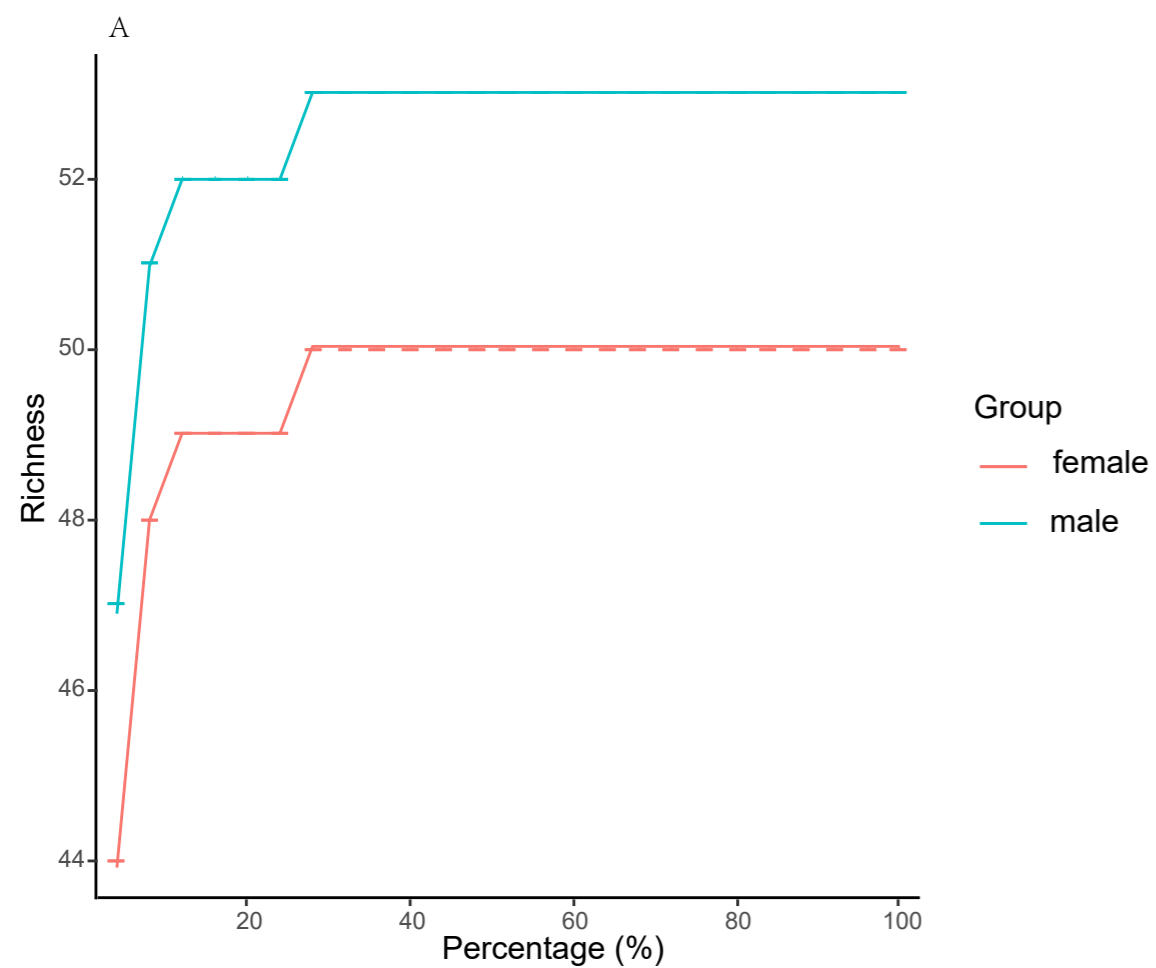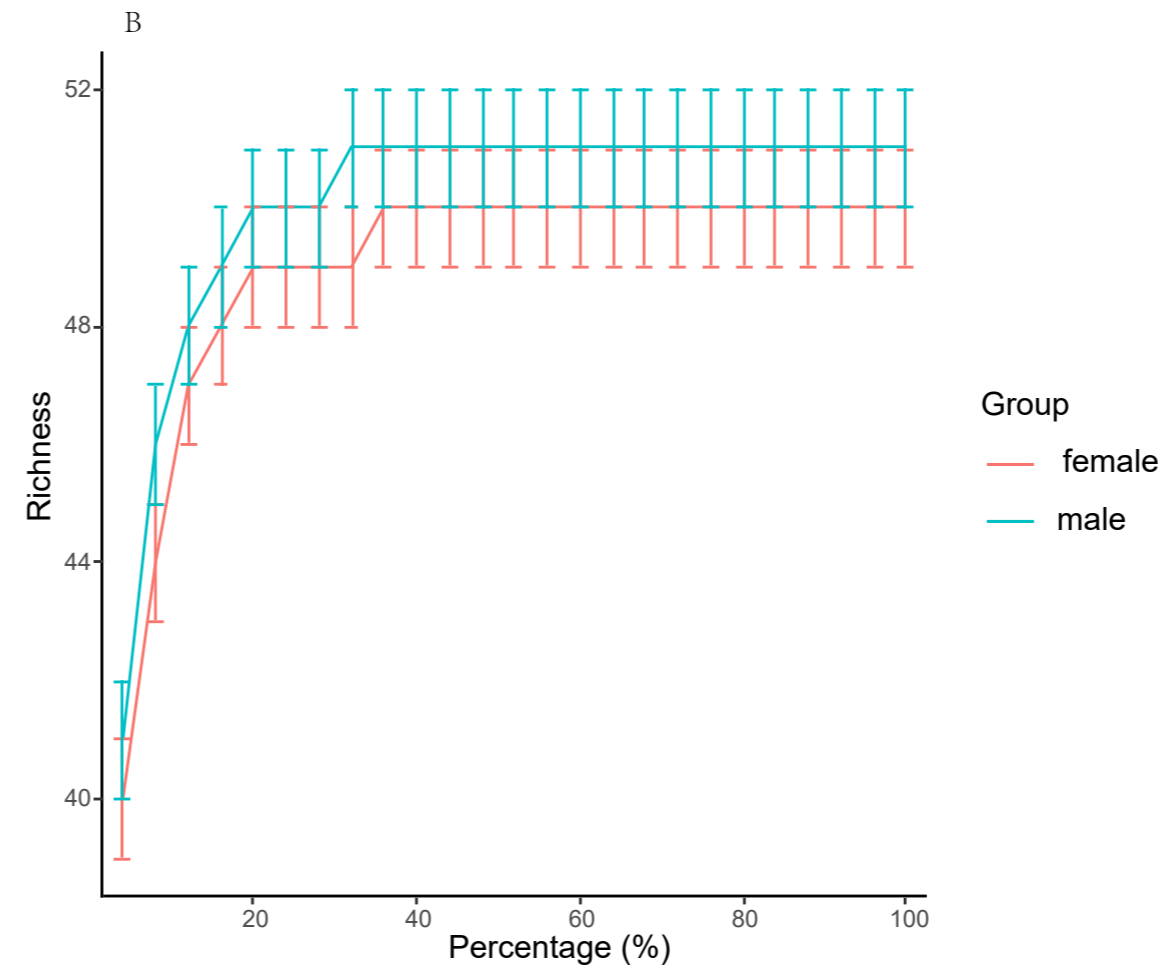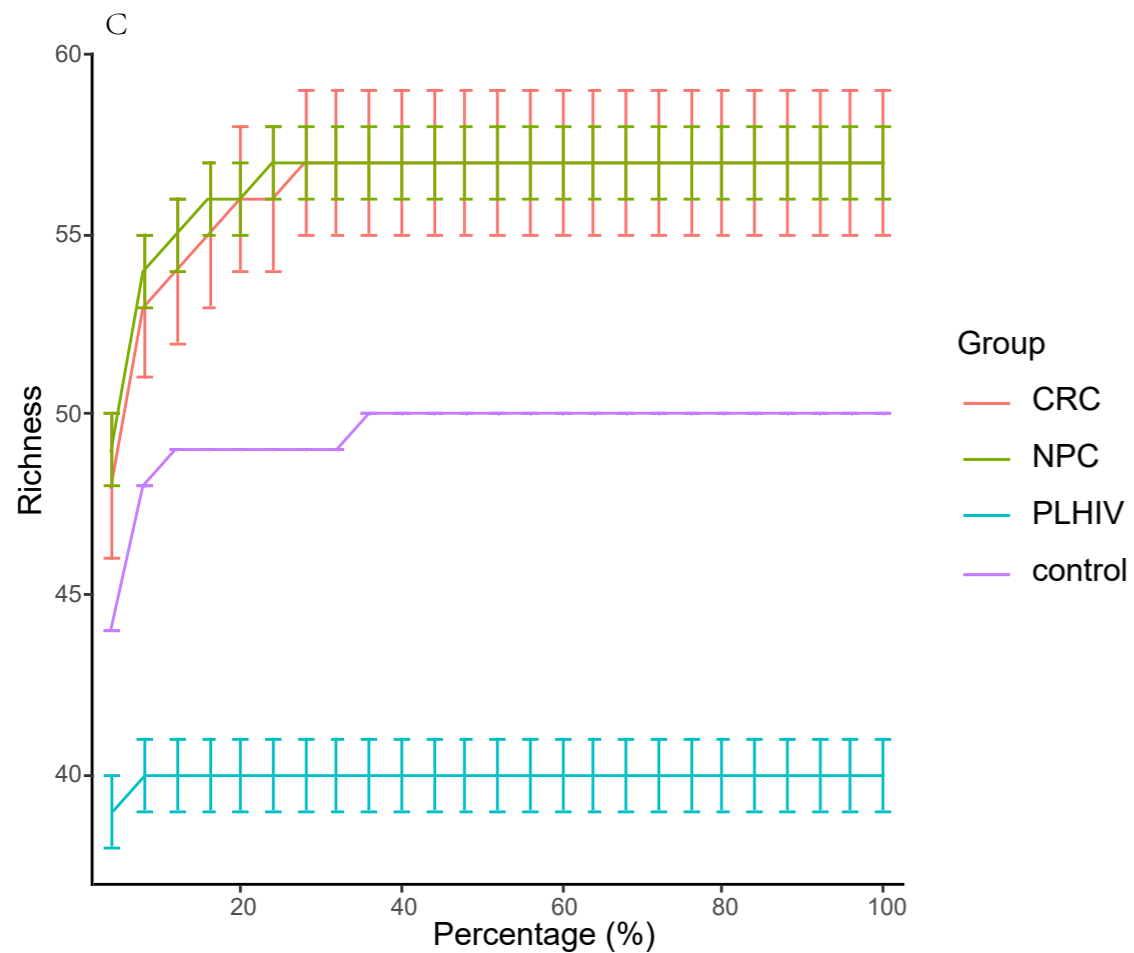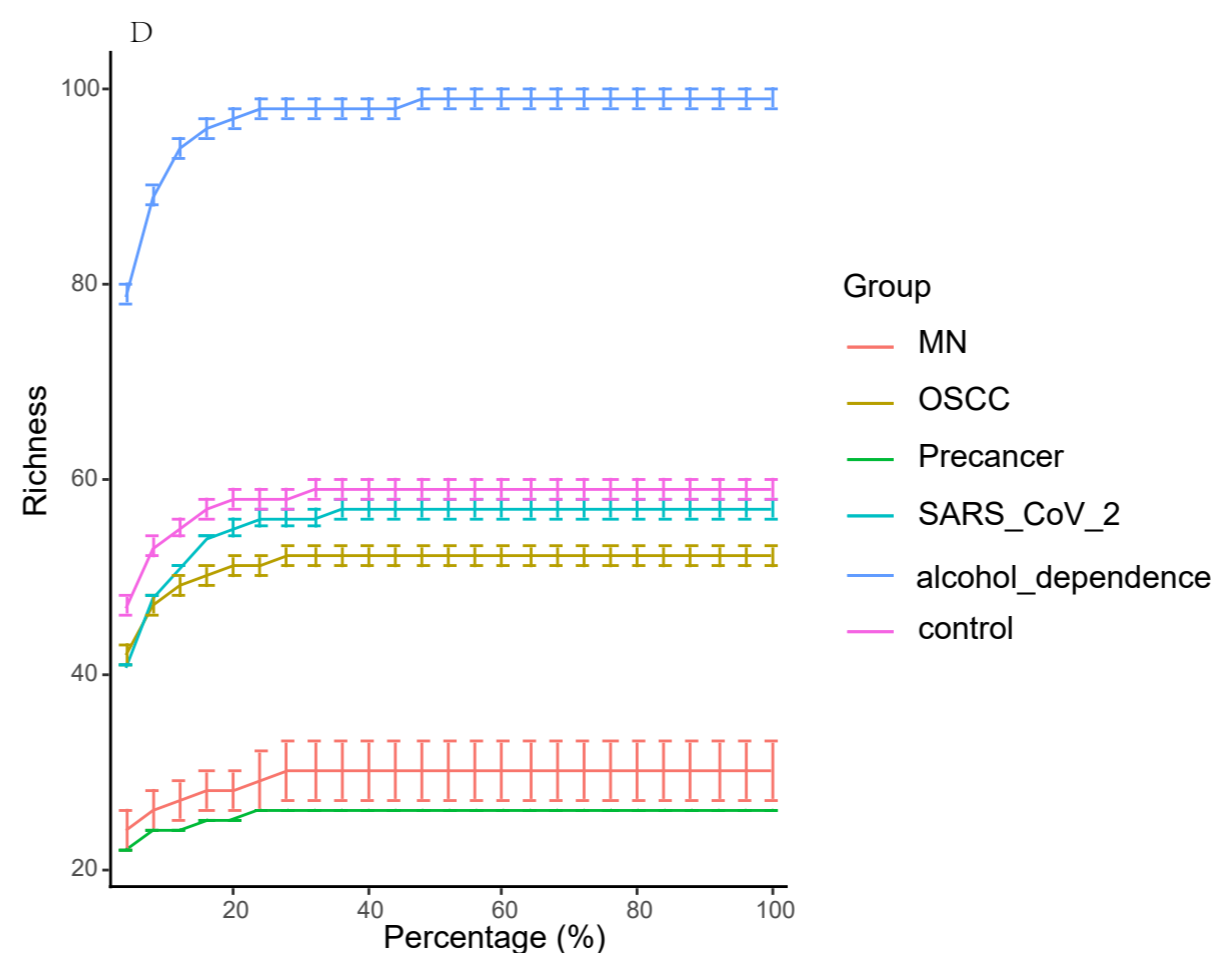

Supplement: Supplementary Figure 2 — (A) species dilution curves of V3-V4 region’s negative controls. (B) species dilution curves of V4 region’s negative controls. (C) species dilution curves of V3-V4 region. (D) species dilution curves of V4 region. Different colors represent different sources. [file DataSheet2.pdf]

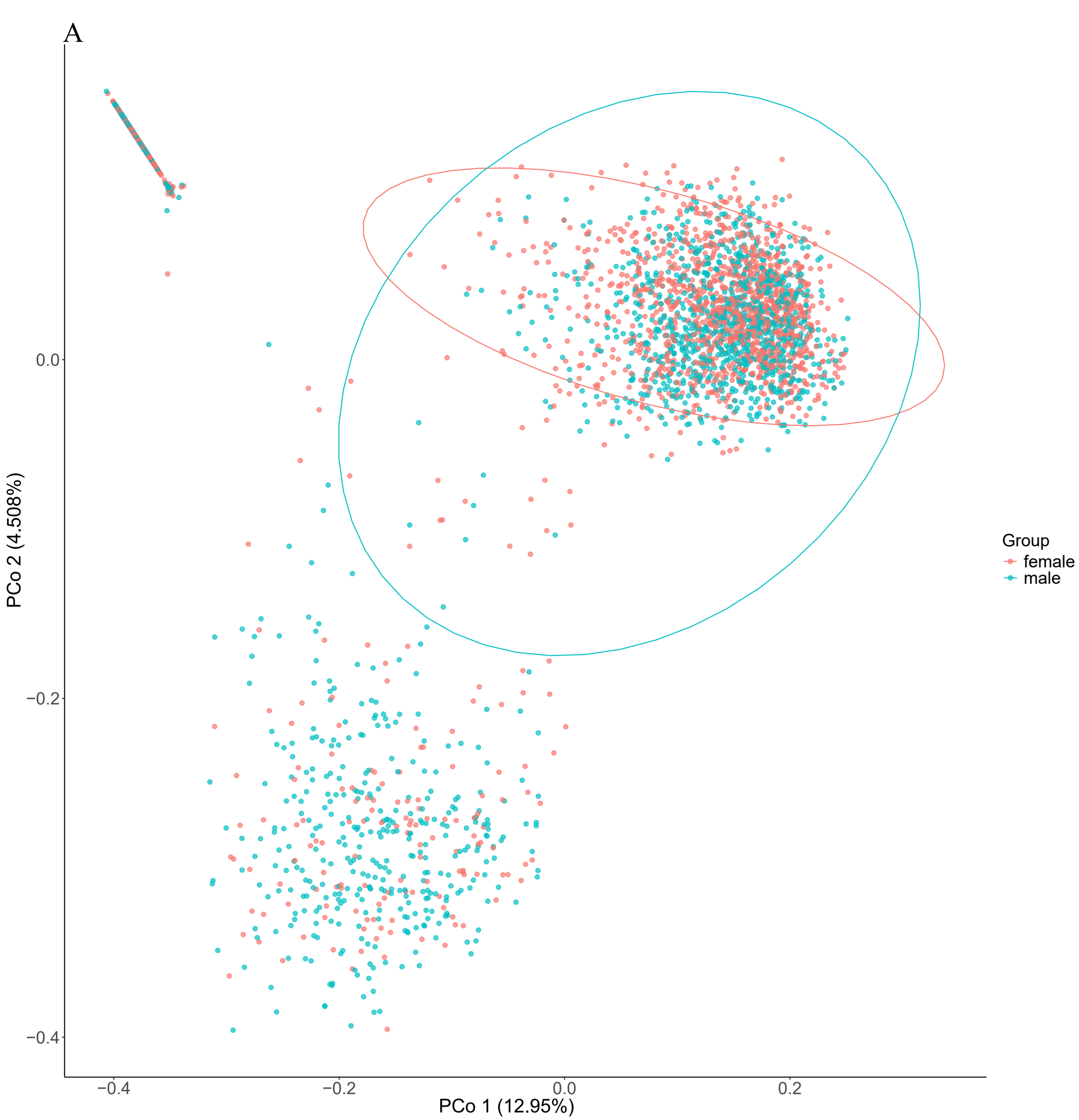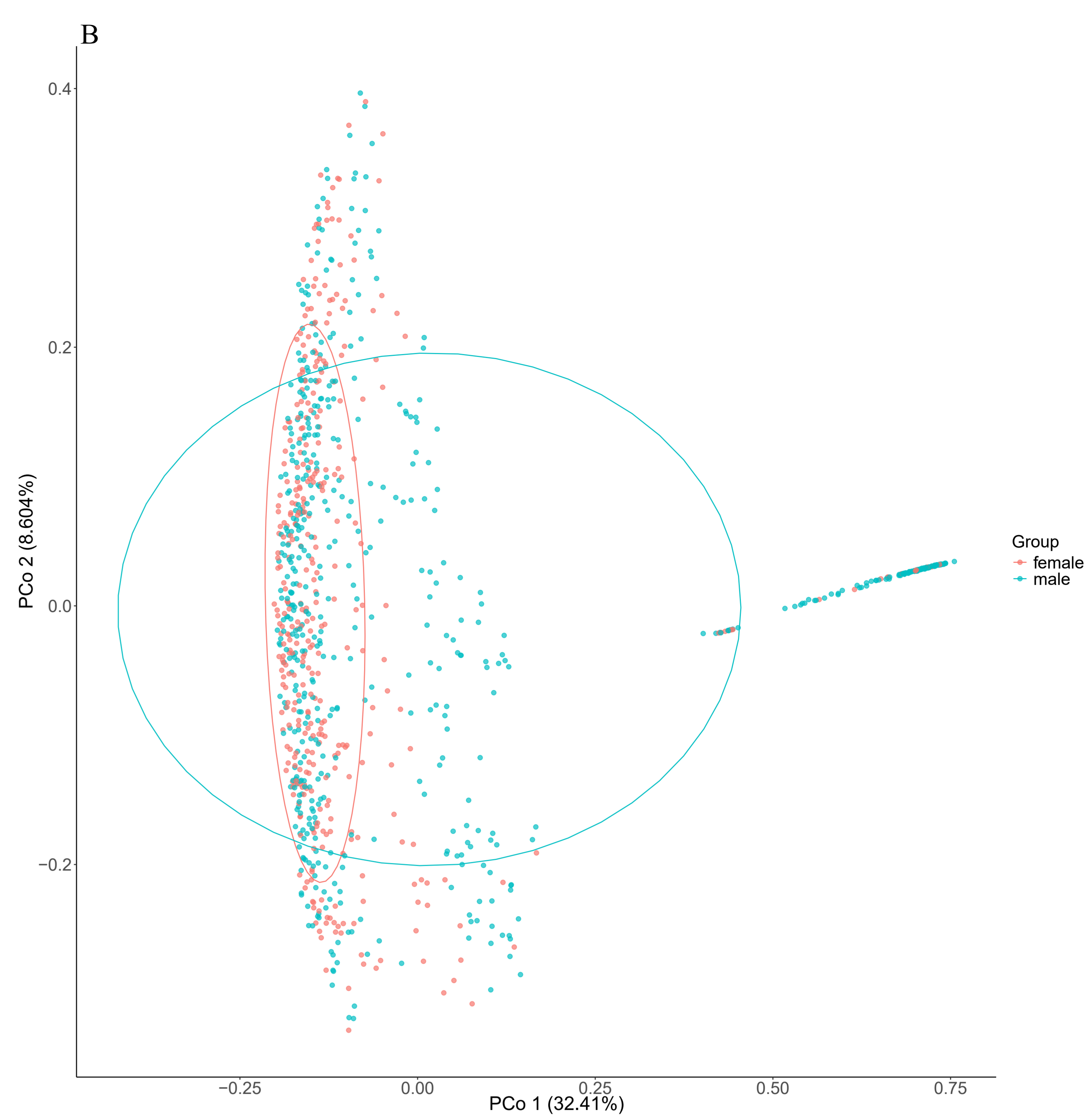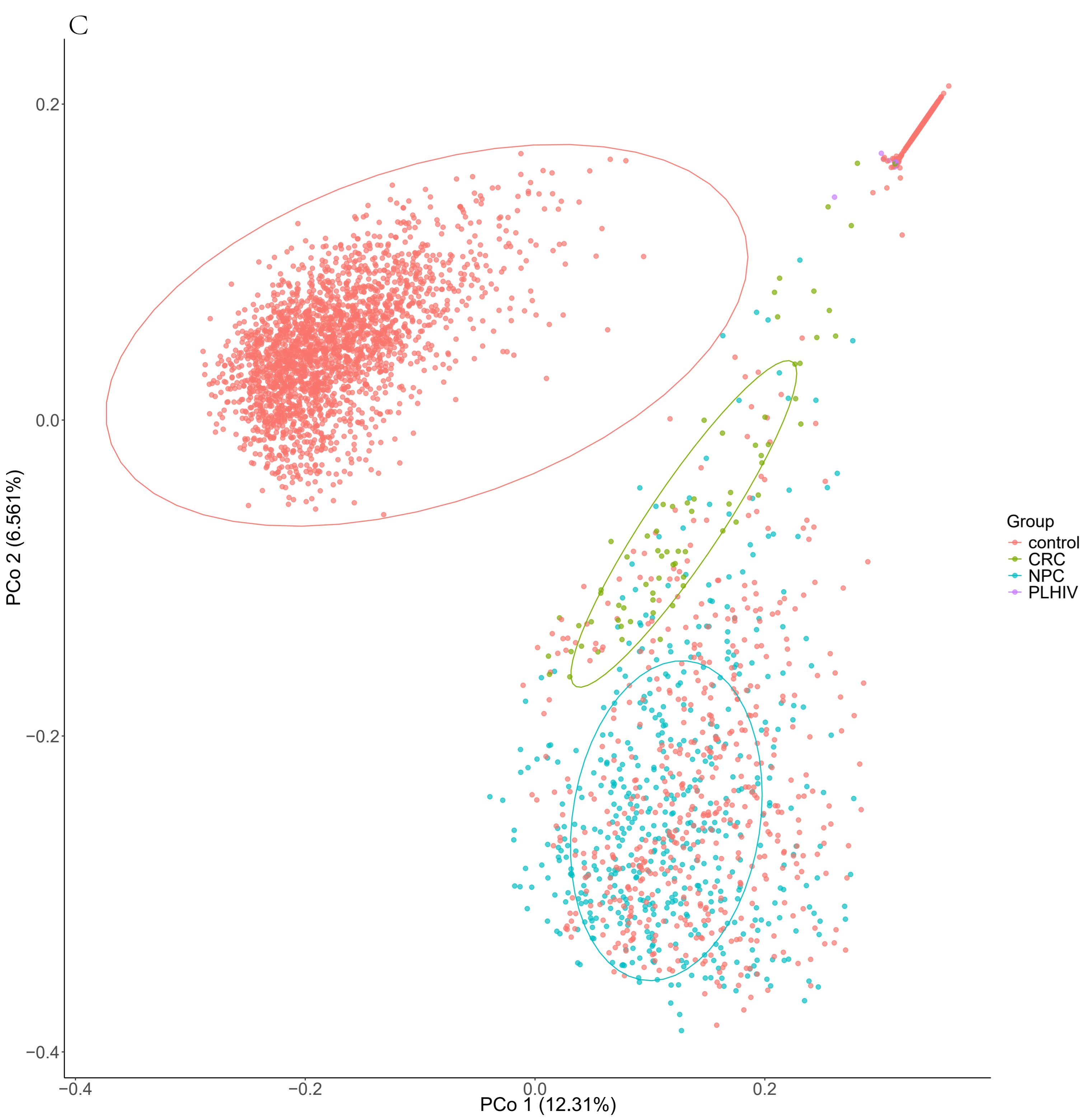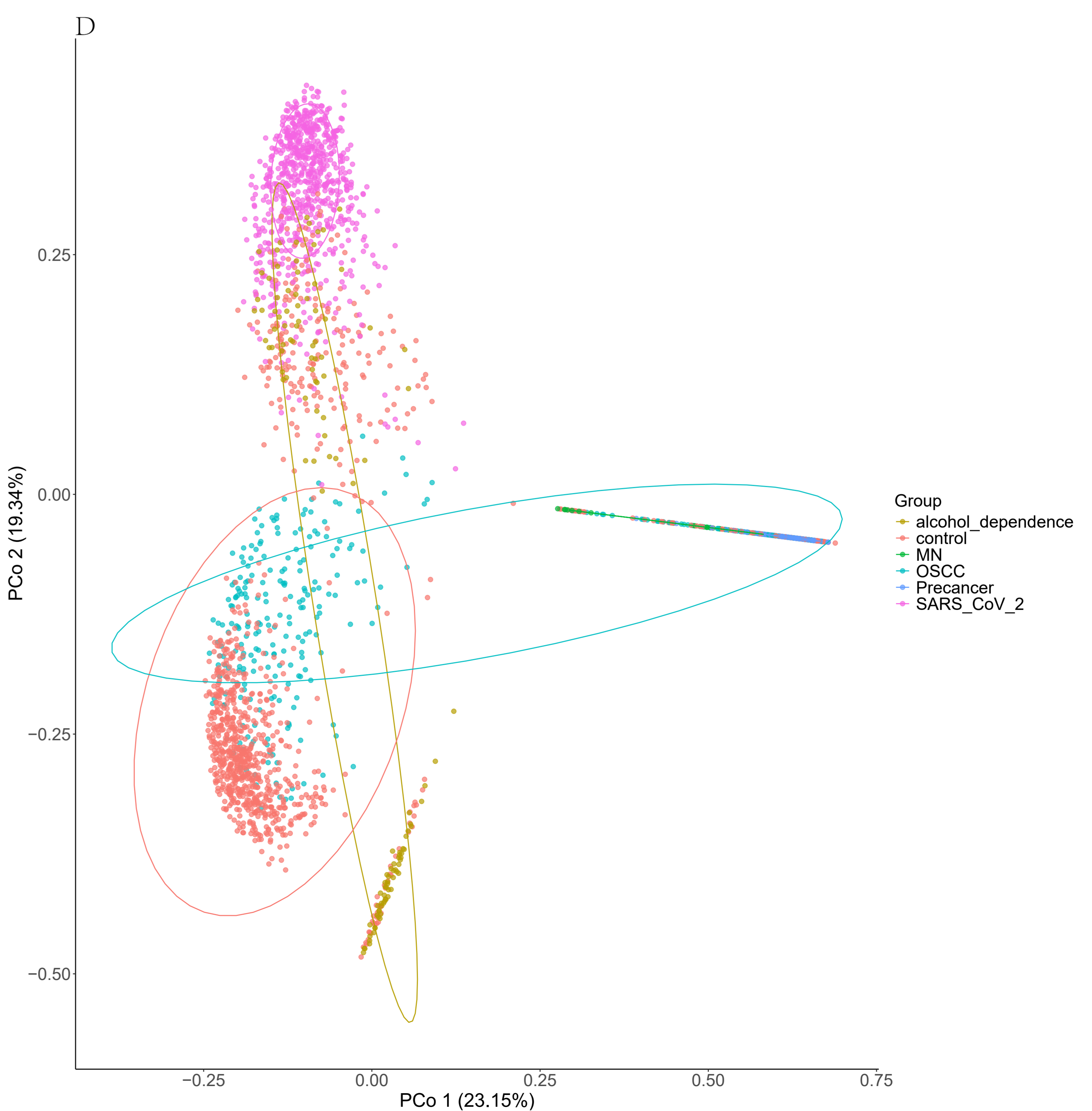

Supplement: Supplementary Figure 3 — (A) PCoA analysis of V3-V4 region’s negative controls. (B) PCoA analysis of V4 region’s negative controls. (C) PCoA analysis of V3-V4 region. (D) PCoA analysis of V4 region. Different colors represent different sources, Each point represents a sample. [file DataSheet3.pdf]

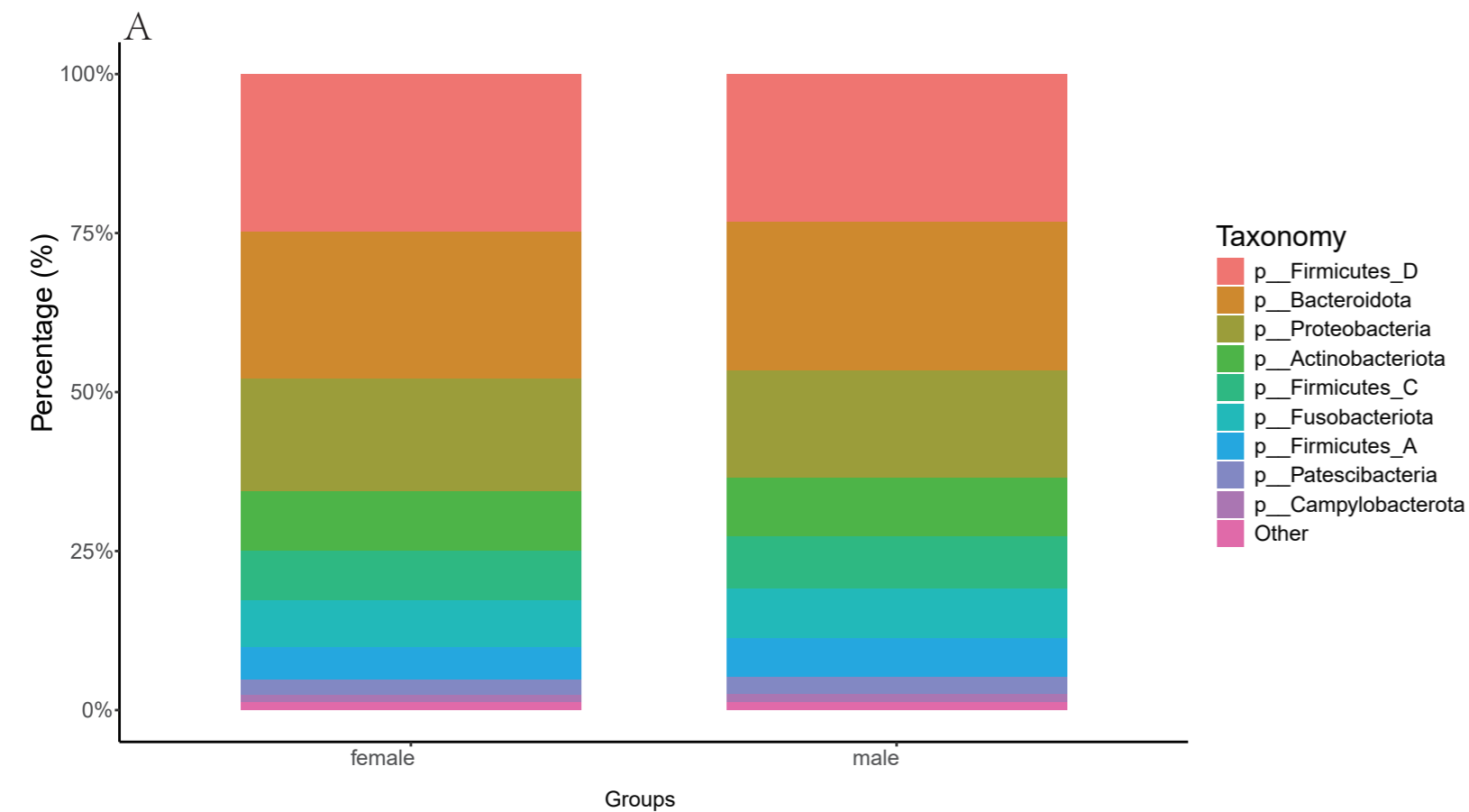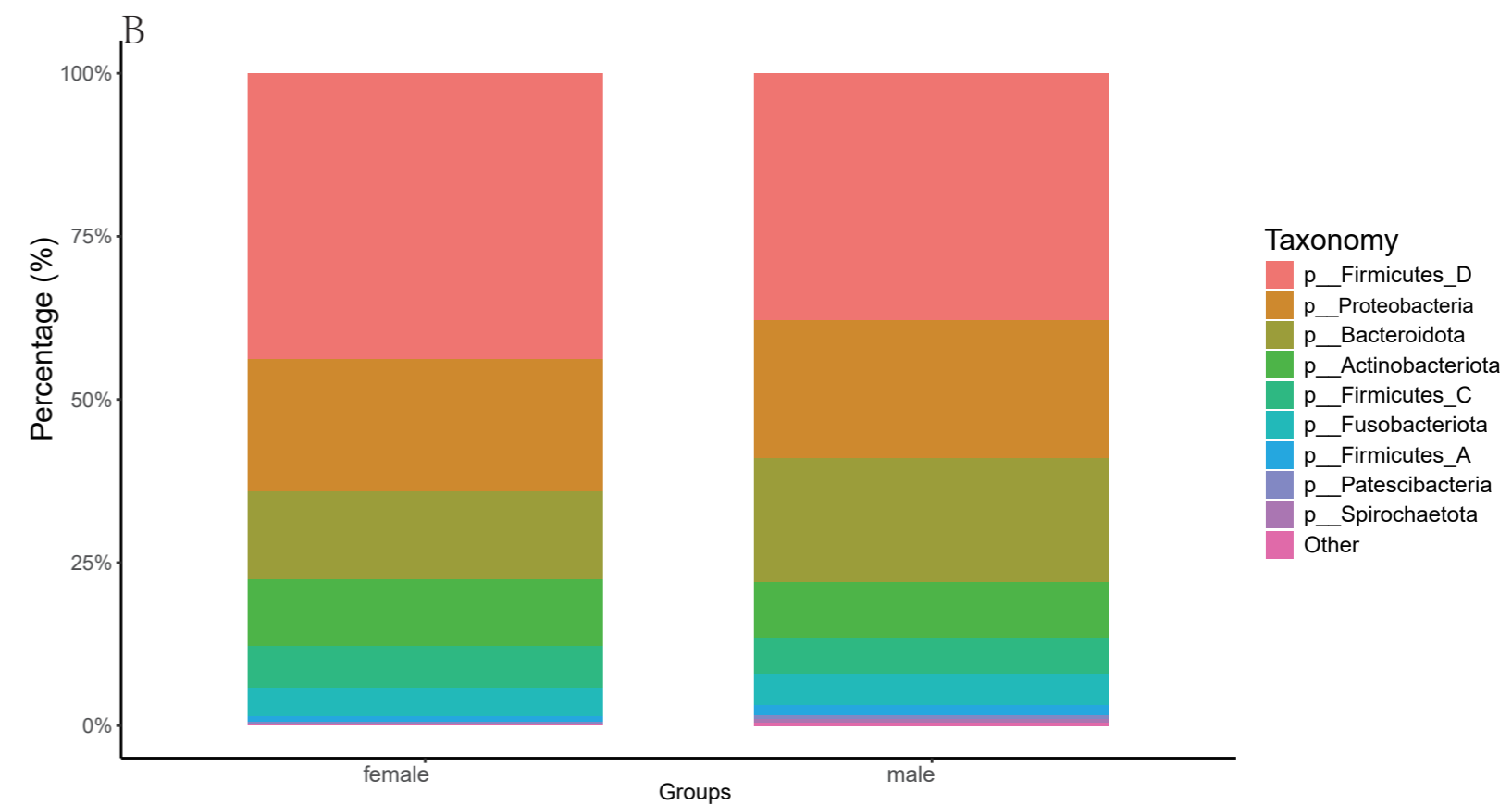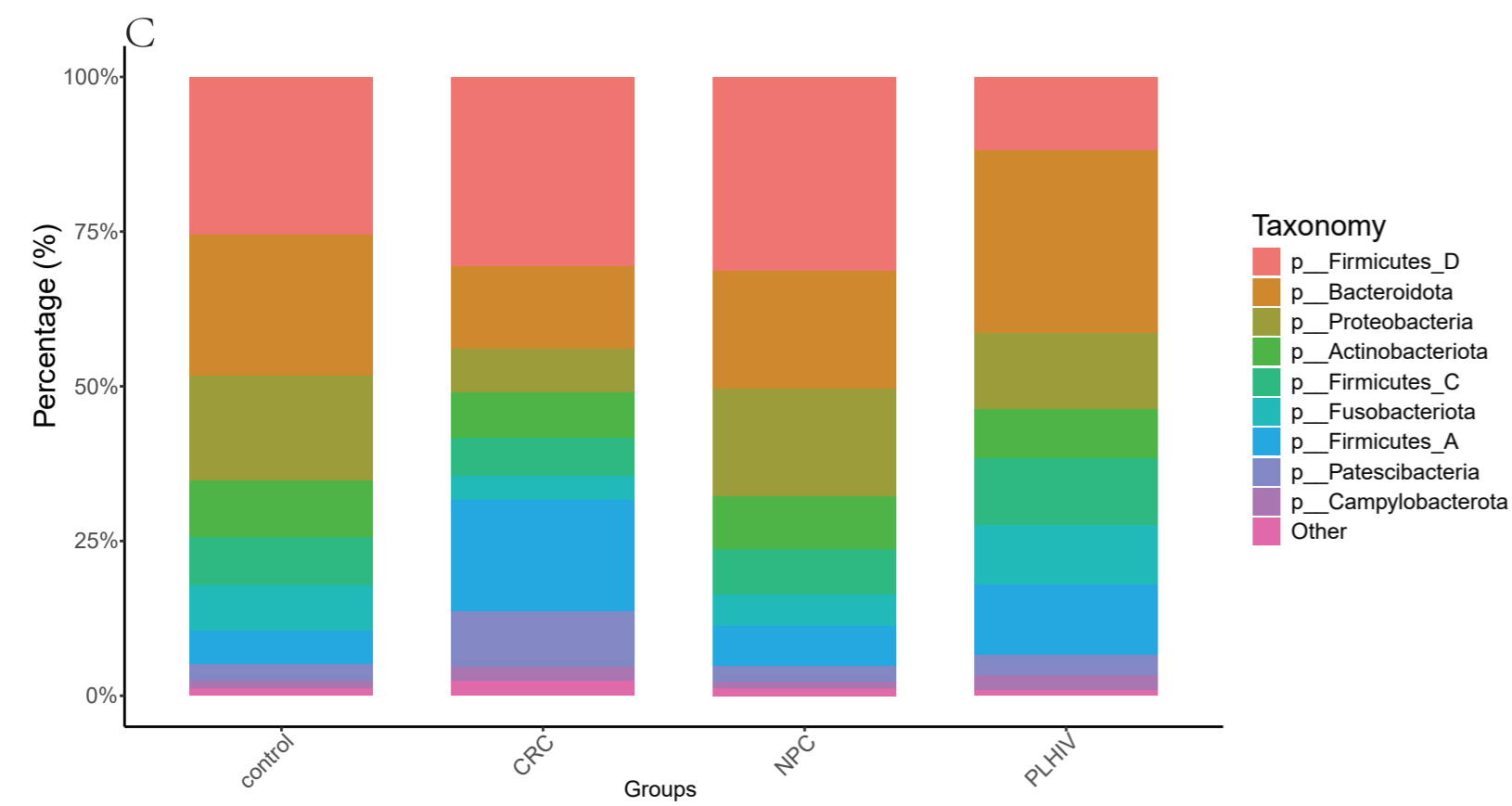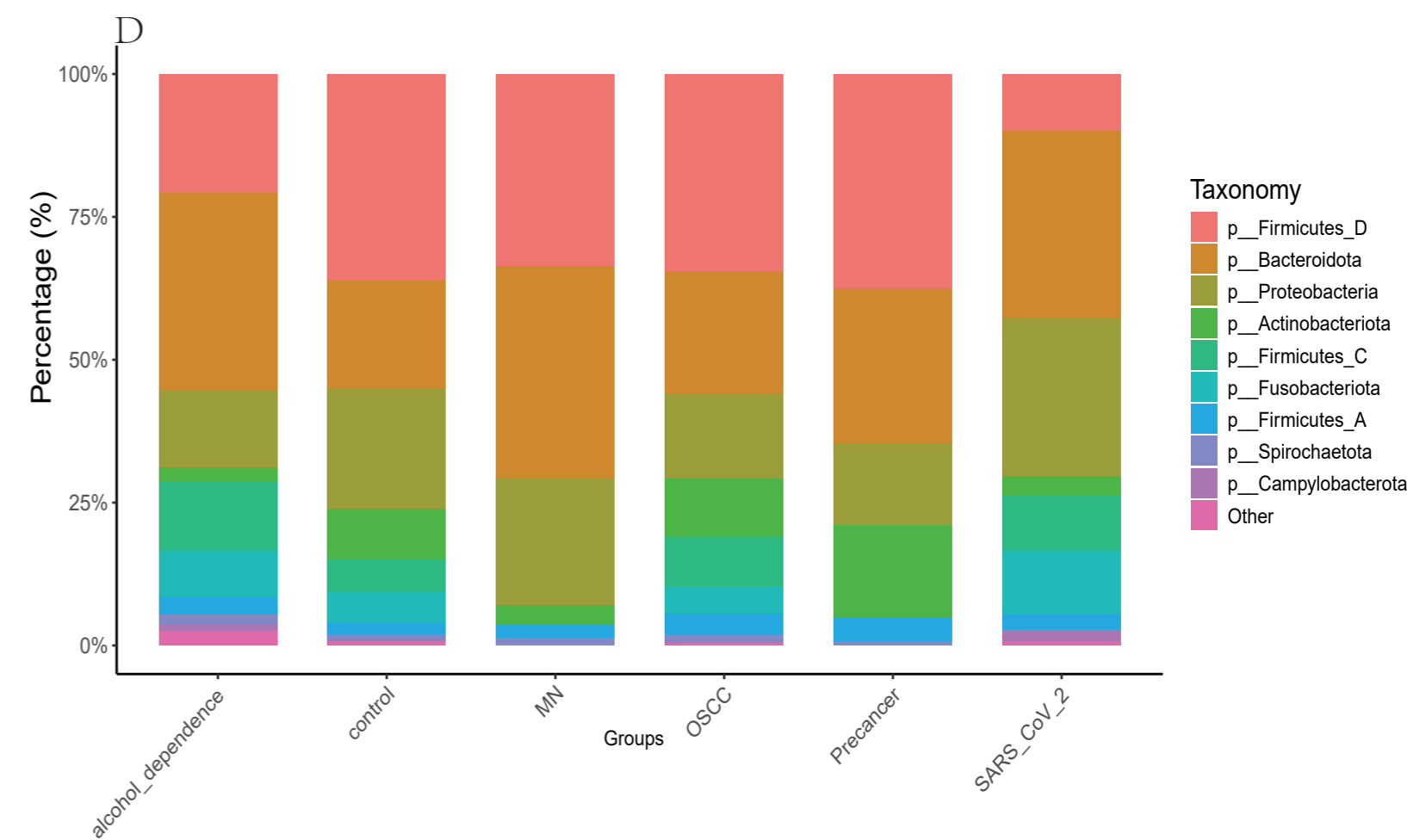

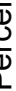

Supplement: Supplementary Figure 4 — (A) Species composition at the phylum level in V3-V4 region’s negative controls. (B) Species composition at the phylum level in V4 region’s negative controls. (C) Species composition at the phylum level in V3-V4 region. (D) Species composition at the phylum level in V4 region. Different colors represent different bacteria. [file DataSheet4.pdf]

A

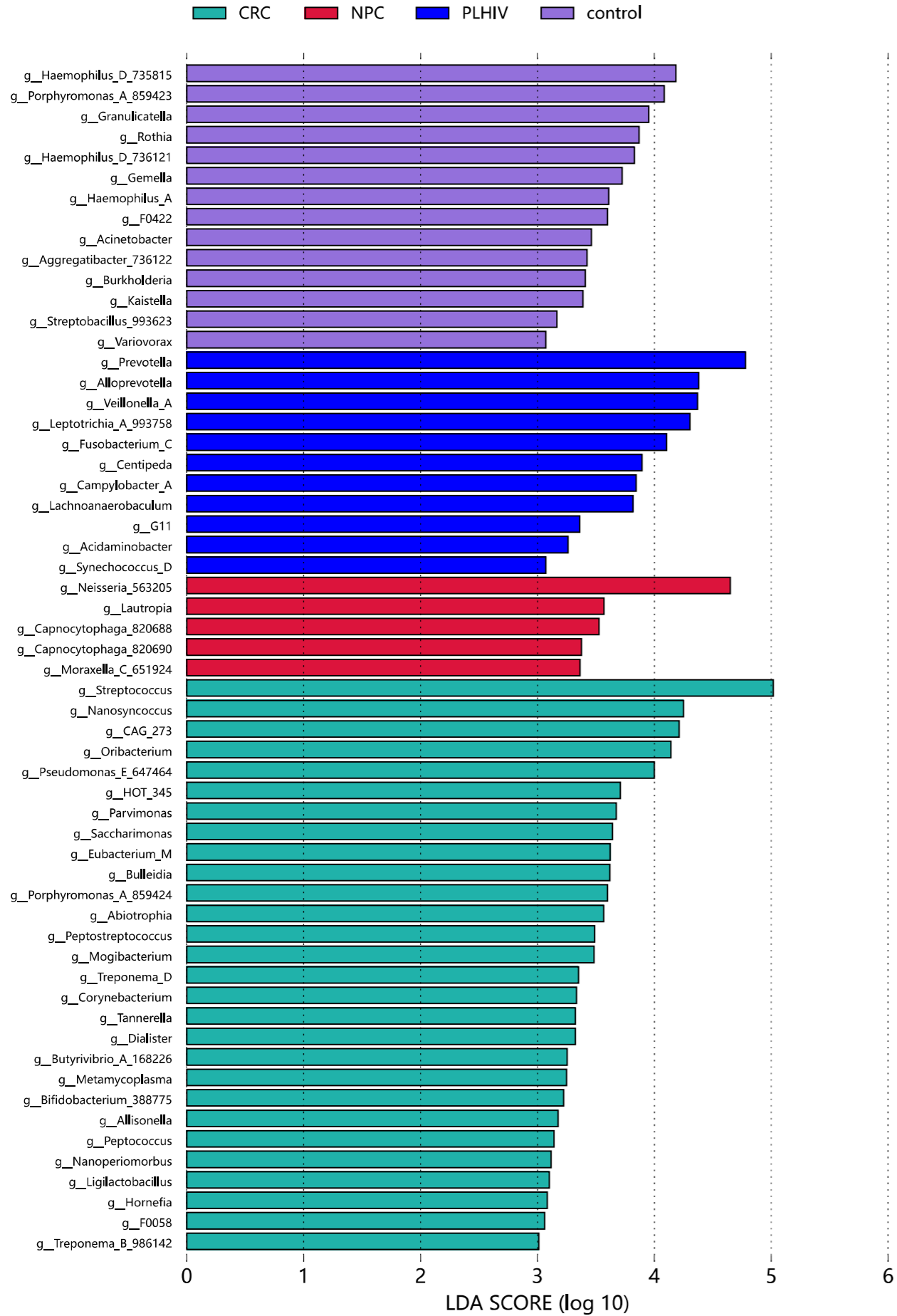

B

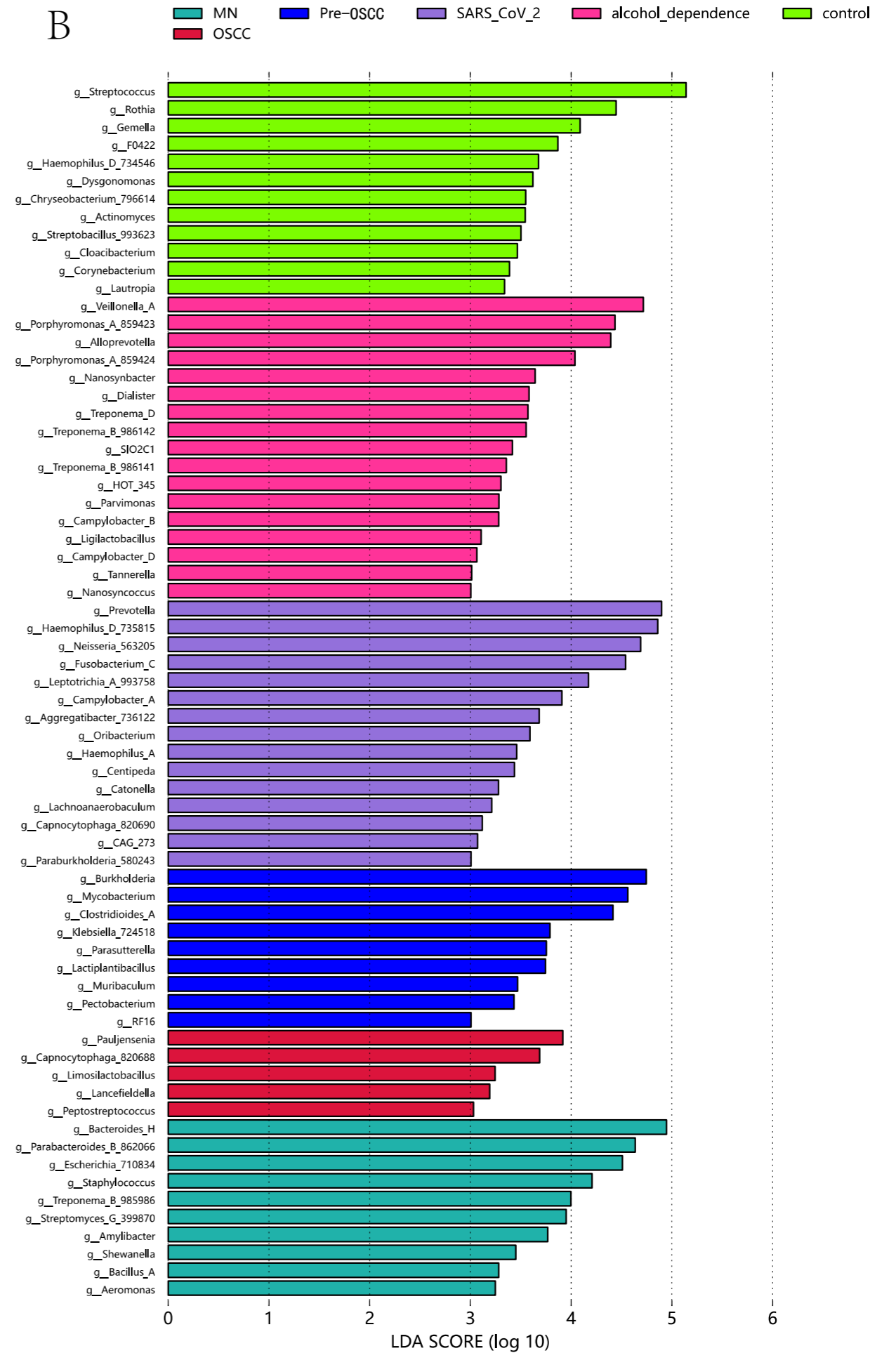

Supplement: Supplementary Figure 6 — (A) LEfSe of V3-V4 region. (B) LEfSe of V4 region. Different colors represent different bacteria. [file DataSheet6.pdf]

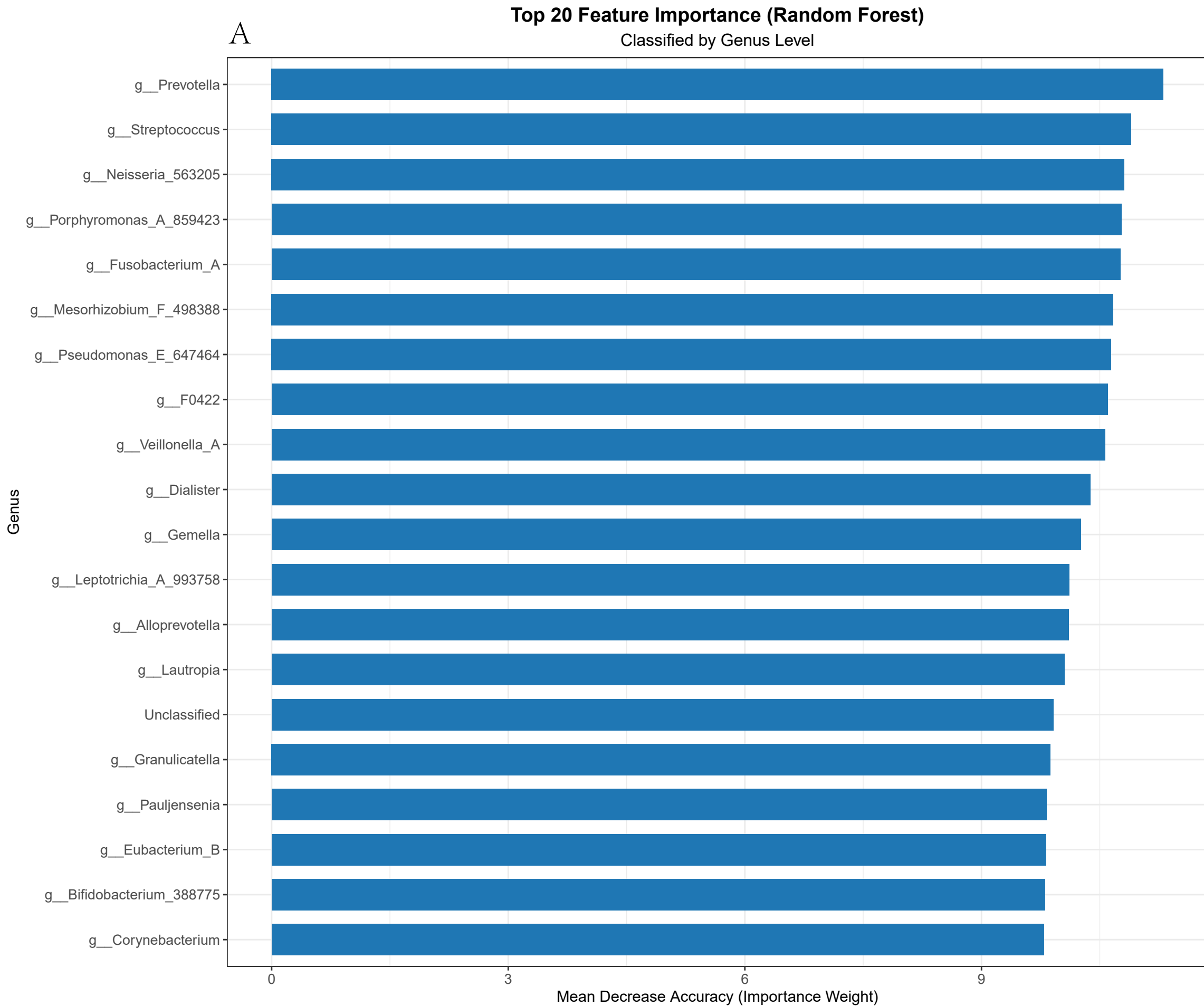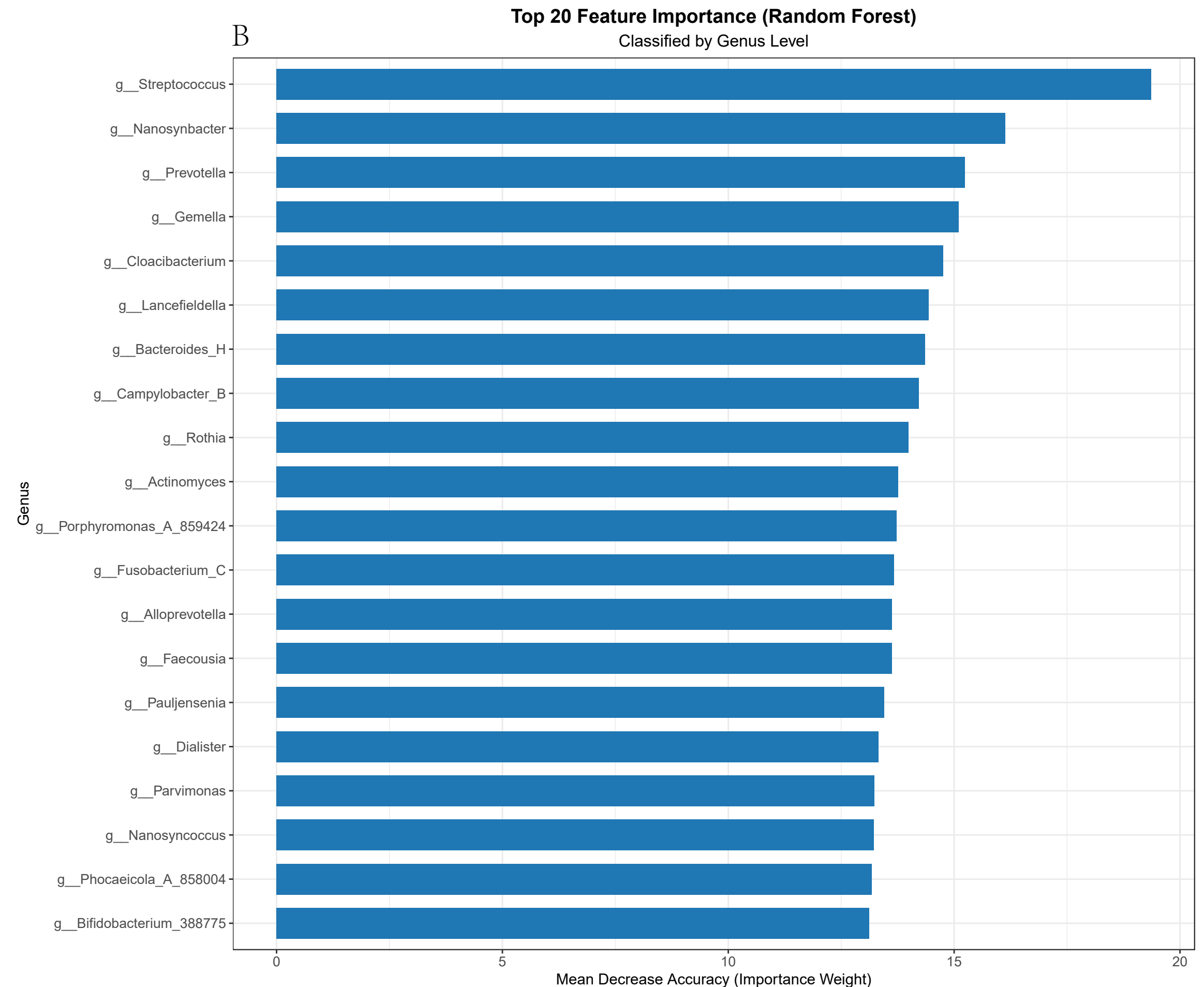

Supplement: Supplementary Figure 7 — (A) Top 20 feature of V3-V4 region. (B) Top 20 feature of V3-V4 region. [file DataSheet7.pdf]
