# Supplementary material for: Salivary microbial meta-analysis reveals gender differences in oral microbiota, core microbiota, and molecular markers
Source: Front Cell Infect Microbiol. 2026 Apr 15;16:1796284. doi: 10.3389/fcimb.2026.1796284 (PMC13125147; doi:10.3389/fcimb.2026.1796284)
Supplement: Supplementary Figure 5 — Venn diagram of core microbiota. [file DataSheet5.pdf]

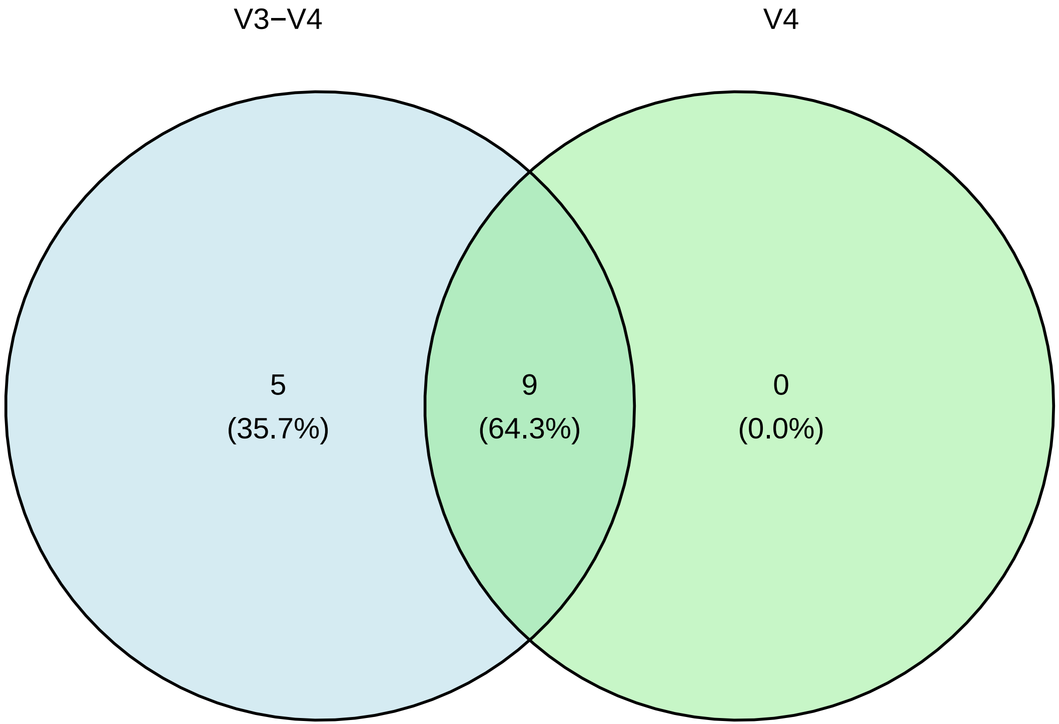

core:
g__Streptococcus
g__Haemophilus_D_735815
g__Prevotella
g__Granulicatella
g__Veillonella_A
g__Neisseria_563205
g__Gemella
g__Porphyromonas_A_859423
g__Rothia

V3−v4_specific:
g__Fusobacterium_C
g__Leptotrichia_A_993758
g__Pauljensenia
g__Alloprevotella
g__Actinomyces

v4_specific:
